# Analysis of novel caudal hindbrain genes reveals different regulatory logic for gene expression in rhombomere 4 versus 5/6 in embryonic zebrafish

**DOI:** 10.1186/s13064-018-0112-y

**Published:** 2018-06-26

**Authors:** Priyanjali Ghosh, Jennifer M. Maurer, Charles G. Sagerström

**Affiliations:** Department of Biochemistry and Molecular Pharmacology, University of Massachusetts Medical School, 364 Plantation St/LRB815, Worcester, MA USA

**Keywords:** Hindbrain, Rhombomere, Gene regulatory network, Retinoic acid, Fibroblast growth factors, PG1 *hox*, *hnf1ba*, *Valentino*

## Abstract

**Background:**

Previous work aimed at understanding the gene regulatory networks (GRNs) governing caudal hindbrain formation identified morphogens such as Retinoic Acid (RA) and Fibroblast growth factors (FGFs), as well as transcription factors like *hoxb1b, hoxb1a, hnf1ba,* and *valentino* as being required for rhombomere (r) r4-r6 formation in zebrafish. Considering that the caudal hindbrain is relatively complex – for instance, unique sets of neurons are formed in each rhombomere segment – it is likely that additional essential genes remain to be identified and integrated into the caudal hindbrain GRN.

**Methods:**

By taking advantage of gene expression data available in the Zebrafish Information Network (ZFIN), we identified 84 uncharacterized genes that are expressed in r4-r6. We selected a representative set of 22 genes and assayed their expression patterns in *hoxb1b, hoxb1a, hnf1b,* and *valentino* mutants with the goal of positioning them in the caudal hindbrain GRN*.* We also investigated the effects of RA and FGF on the expression of this gene set. To examine whether these genes are necessary for r4-r6 development, we analyzed germline mutants for six of the genes (*gas6, gbx1, sall4, eglf6, celf2*, and *greb1l*) for defects in hindbrain development.

**Results:**

Our results reveal that r4 gene expression is unaffected by the individual loss of *hoxb1b, hoxb1a* or RA, but is under the combinatorial regulation of RA together with *hoxb1b*. In contrast, r5/r6 gene expression is dependent on RA, FGF, *hnf1ba* and *valentino* – as individual loss of these factors abolishes r5/r6 gene expression. Our analysis of six mutant lines did not reveal rhombomere or neuronal defects, but transcriptome analysis of one line (*gas6* mutant) identified expression changes for genes involved in several developmental processes – suggesting that these genes may have subtle roles in hindbrain development.

**Conclusion:**

We conclude that r4-r6 formation is relatively robust, such that very few genes are absolutely required for this process. However, there are mechanistic differences in r4 versus r5/r6, such that no single factor is required for r4 development while several genes are individually required for r5/r6 formation.

**Electronic supplementary material:**

The online version of this article (10.1186/s13064-018-0112-y) contains supplementary material, which is available to authorized users.

## Background

During vertebrate embryogenesis, cells in the presumptive hindbrain are sorted into seven molecularly and neuroanatomically unique segments called rhombomeres (r1-r7). Segmentation creates regional diversity along the anterior-posterior (A-P) axis of the hindbrain and sets the stage for proper neuronal development and cranial neural crest migration [1–3]. The vertebrate hindbrain is responsible for regulating complex processes such as breathing, heartbeat, circulation, wakefulness and cranio-facial development [4, 5]. Precise hindbrain patterning is critical for the development of a fully functional central nervous system (CNS) and defects in this process have been linked to neurological disorders like autism [6, 7]. Thus, it is crucial to understand the regulatory mechanisms underlying hindbrain formation.

In the zebrafish, the earliest factors to be expressed in the hindbrain include the posteriorizing morphogens Retinoic acid (RA) and Fibroblast growth factors (FGFs) [8–13]. Around 6 h post fertilization (hpf), RA is detected in the posterior paraxial mesoderm from where it diffuses throughout the neural tube creating a spatial gradient with the caudal hindbrain being exposed to higher concentrations of RA [14–19]. FGF signaling (*fgf3* and *fgf8a*) is detected as early as 8hpf in the presumptive r4 [19, 20]. Expression of these morphogens initiates the division of the hindbrain primordium into rostral and caudal regions. The subsequent expression of transcription factors (TFs) like *hoxb1b*, *hoxb1a*, *hnf1ba*, *valentino* and *krox20* further subdivides the caudal hindbrain into r4, r5 and r6. Expression levels of these factors have been manipulated to gain insight into how they interact with one another. For example, loss of RA signaling results in posterior expansion of r4 gene expression domains (e.g. *hoxb1a*), and reduced expression of r5/r6 genes like *hnf1ba* and *valentino* [16, 17, 21]. Similarly, combined knockdown of Fgf8 and Fgf3 leads to the loss of *krox20* and *valentino* expression in r5 [19, 20]. Accordingly, mutations in the *hnf1ba* and *valentino* genes cause mis-patterning of r5/r6, with the posterior expansion of *hoxb1a* and *efnb2a,* loss of *krox20* expression in r5, and loss of abducens motor neurons [22, 23]. Germline mutants for *hoxb1b* have a smaller r4, with mis-patterned cranial motor neurons, and partial loss of Mauthner neuron formation [24–26]. Similar neuronal defects are also seen in *hoxb1a* mutants [24, 26]. These results have led to a model for the gene regulatory network (GRN) underlying caudal hindbrain formation. This model posits that RA triggers caudal hindbrain patterning by initiating the expression of *hoxb1b* (in r4-r7) and *hnf1ba* (in r5/r6) [16]. *hoxb1b* turns on the expression of *hoxb1a*, which sustains its own expression through an autoregulatory loop. Thus, the expression of the paralog group 1 (PG1) *hox* genes leads to the formation and specification of r4 [24–29]. *hnf1ba* together with FGF signaling activates *valentino* expression in r5/r6 [23, 30], which in turn regulates *krox20* expression in r5. [20, 31]. Thus, within the caudal hindbrain GRN, there seems to be two relatively linear pathways that regulate r4 and r5/r6 formation, while cross-talk between these pathways maintains the integrity of each rhombomere.

Despite numerous genetic approaches – initially using chemical (ENU; [32–34] [22]) and retroviral [35–37] mutagens, but more recently also applying TILLING [38], Zinc Finger Nucleases [24] and TALENs [24] – RA, FGF, *hoxb1b, hoxb1a, hnf1ba,* and *valentino* remain the key factors required for caudal hindbrain formation in zebrafish. This is a surprisingly small number considering that other GRNs associated with developmental processes (e.g. germ layer differentiation in sea urchin [39], embryonic development in *C. elegans* [40], pancreas formation [41], mouse neural tube specification [42] and zebrafish endoderm formation [43]) are more complex [41] — implying that there may be additional genes acting within the caudal hindbrain GRN. It is possible that shortcomings of genetic screens (such as the necessary bias towards readily detectable phenotypes [32, 33, 37, 44]) may have overlooked other genes acting in the caudal hindbrain GRN. Indeed, induction of *Hoxb1* and *Hoxa1* in murine embryonic stem (ES) cells [45–48], MO-knockdown of *hoxb1b* and *hoxb1a* [27, 49, 50], and overexpression of *hoxb1b* and *hoxb1a* [51, 52] has identified additional genes expressed in the caudal hindbrain. While a few of these genes may have roles in the hindbrain (e.g. migration of neural crest cells and neuronal patterning and differentiation; [48, 50]), most have not been assayed functionally. Hence, the goal of our study was to identify novel regulators required for caudal hindbrain development and position them within the GRN governing caudal hindbrain formation.

We reasoned that potential regulators should be expressed in r4-r6 at early stages of development—similar to the TFs *hoxb1b, hoxb1a, hnf1ba, valentino* and *krox20*. To find such genes, we analyzed the gene expression data deposited in the Zebrafish Information Network (ZFIN) and identified 107 genes that are expressed in r4, r5 and r6 during the first 24 h of zebrafish development. The majority (*n* = 84) of these 107 genes have not been extensively characterized previously, suggesting that they may represent novel regulators of caudal hindbrain formation. To test this, we selected 22 representative genes and assayed their expression patterns in zebrafish mutants for *hoxb1b, hoxb1a, hnf1ba,* and *valentino.* We also investigated the effects of the morphogens RA and FGF on the expression of these genes. Lastly, we assayed germline mutants for six of the genes (*gas6, gbx1, sall4, eglf6, celf2*, and *greb1l*) for defects in hindbrain and neuronal patterning. Strikingly, our results show that genes expressed in r4 are not affected by the loss of *hoxb1b* or *hoxb1a*. Loss of RA and FGF signaling also does not affect r4 gene expression (except for *dusp2, dusp6, spry1, fgf3* and *fgf8* - which are components of the FGF signaling pathway itself). Instead, we find that all tested r4 genes are under the combinatorial regulation of RA and *hoxb1b*. Furthermore, we observe that *hoxb1a* (either directly or indirectly) represses the expression of *gbx1* in r4, revealing a novel relationship between *hoxb1a* and *gbx1*. In contrast to the situation in r4, r5/r6 gene expression requires each of RA, FGF, *hnf1ba* and *valentino* – whereby loss of any one of those four factors blocks r5/r6 gene expression. Lastly, analysis of hindbrain and neuronal markers revealed that mutations in *gas6, gbx1, sall4, eglf6, celf2*, and *greb1l* are not sufficient to cause detectable developmental defects in the caudal hindbrain. Nevertheless, transcriptome analysis of *gas6* mutants identified expression changes in many genes involved in a variety of developmental processes, indicating that these mutants may have very subtle phenotypes. In summary, by positioning 22 novel genes into the caudal hindbrain GRN, we demonstrate that gene regulation in r4 is robust with no single gene being essential, whereas r5/r6 gene expression is susceptible to disruption of either RA, FGF, *hnf1ba* or *valentino* function. We also identify novel interactions between r4 and r5/r6 genes – highlighting the importance of cross-talk between the two gene-sets in maintaining the specific molecular identity of each rhombomere.

## Methods

### Zebrafish care

Wildtype (WT) and mutant zebrafish embryos were collected through natural matings. All embryos were staged according to previously described morphological criteria [53]. All zebrafish lines were raised in the University of Massachusetts Medical School Zebrafish Facility.

### In situ hybridization

Embryos were collected at various timepoints between 11hpf and 24hpf and were fixed in 4% paraformaldehyde and stored in 100% methanol at − 20°C. In situ hybridization (ISH) was performed as previously described and was followed by a color reaction using NBT/BCIP or INT/BCIP in 10% polyvinyl alcohol [54]. Synthesis of RNA probes for the genes *dusp6*, *dusp2*, *krox20*, *hoxb1a, fgf3*, *fgf8* and *valentino* has been previously described [55]. 800-1000 bp of coding sequence for the genes *pax2, spry1*, *hoxd4a, dm20, efnb2a, sall4, greb1l, egfl6, hoxb2a, engrailed1b, irx7, meis1a, tox3, sema3fb, mpz, gas6, hoxb3a, hoxa3, isl1/2, neurod6b, atoh1b, olig4* and *nr2f2* were cloned and used for probe synthesis. The *ccnjl, cefl2, col15a1b* and *gbx1* probes were purchased from the Zebrafish International Resource Center (ZIRC). Each ISH experiment included a positive control of wildtype embryos stained with the same probe as the experimental samples. Control and experimental ISH reactions were stopped when control embryos reached optimal staining. Panels shown in each figure were not necessarily processed on the same day.

### Immunostaining

For whole-mount immunostaining, embryos were fixed in 4% paraformaldehyde/8% sucrose/1× PBS. Fluorescent antibody staining was performed as described previously [56]. Primary antibodies were used to detect Mauthner neurons (3A10; 1:100; Developmental Studies Hybridoma Bank [DSHB]), and Abducens motor neurons (mouse anti-Zn8; 1:1000; DSHB). The secondary antibody used was goat anti-mouse Alexa Fluor 488 (1:200; Molecular Probes A11001).

### Imaging

Embryos between 11hpf and 19hpf were suspended in 3% methyl cellulose for imaging. Images were captured using a Leica M165 FC microscope equipped with a Leica DFC310 FX camera. 24 hpf, 48 hpf and 4 days post fertilization (dpf) old embryos were de-yolked and flat-mounted in 70% glycerol for imaging on bridged coverslips. Whole-mount embryos were imaged with a Nikon Eclipse E600 microscope equipped with a Nikon 20× Plan Fluor objective and flat-mounted embryos were imaged with a Zeiss Axiocam 503 color camera. Captured images were cropped and adjusted (limited to contrast and levels) in Adobe Photoshop.

### Pharmacological treatments

A 250 mM stock solution of SU5402 (a competitive inhibitor of the Fgf receptor tyrosine kinase; Calbiochem) and a 1 M stock solution of 4(Diethylamino)-benzaldehyde (DEAB – small molecule inhibitor of RALDH enzyme involved in RA synthesis; Aldrich) was diluted in DMSO and stored in − 20 °C. To block RA signaling, embryos were soaked in 10uM DEAB starting at 4 hpf. The drug was never washed off and embryos were collected and fixed for ISH at 12hpf, 14hpf 16hpf, 19hpf and 24hpf. Similarly, to block FGF signaling, embryos were soaked in 50uM of SU5402 from 7hpf to 12hpf. After which, embryos were thoroughly rinsed in aquarium water [12] and allowed to develop till 12hpf, 14hpf, 16hpf, 19hpf and 24hpf when they were collected and fixed for ISH.

### Design and injection of single-strand guide RNAs for CRISPR/Cas9 mediated genome editing

*gas6* (alleles um296, um297, um298 and um299) and *gbx1* (alleles um300 and um301) mutants were generated using the CRISPR (Clustered Regularly Interspaced Short Palindromic Repeat)/Cas9 genome editing system. Target sites for *gas6* (5’-ATGAGGGAGCTGGTGTGGAGC-3′) and *gbx1* (5’-CCAGATAGT- TTCTACCCCCC-3′) were determined using the CHOPCHOP web tool for genome editing [57]. Oligos containing a T7 promoter sequence, the target sequence, and an additional constant region were created and annealed according to previously described methods [55, 58]. These templates were transcribed in vitro using T7 RNA polymerase (Promega) to generate single-stranded guide RNAs (sgRNAs) for microinjection. A linearized plasmid encoding *cas9* was also transcribed in vitro using the SP6 mMessage mMachine Kit (Ambion). 200 ng each of the sgRNA and *cas9* mRNA were combined and 2-4 ng of this mixture was injected into early 1-cell stage embryos.

### Genotyping zebrafish lines

The zebrafish mutants *valentino*^*b337*^ [22], *hnf1ba*^*hi2169*^ [35], *hoxb1b*^*um197*^ and *hoxb1a*^*um191*^ [24] were genotyped as previously described. The *greb1l*^*sa17608*^*, egfl6*^*sa21615*^*, sall4*^*sa14110*^ and *celf2*^*sa33469*^ lines were identified via TILLING [59] and mutant alleles were ordered from ZIRC. Mutant alleles were genotyped by sequencing PCR products amplified from genomic DNA using primers.

5’-TGTGAAAATTTCCTTGCTGTGT-3′ and 5’-CTGAAGGGCAGAATACGG-3′ for *greb1l*^*sa17608*^, 5’-ATCACAGATCCTGGGACAGC-3′ and 5’-AAAAGCATTGGATGCA- GCTC-3′ for *egfl6*^*sa21615*^, 5’-GGGCATGAGGAGAGTATGGA-3′ and 5’-TCTTTCAG- CCCACTGTCACTC-3′ for *sall4*^*sa14110*^, and 5’-CTTTGTTGGCGACCATTGA-3′ and 5’-AAAGCGACAAAAACAGATTCG-3′ for *celf2*^*sa33469*^. *gbx1* mutants (alleles um300 and um301) were genotyped by Hpy188III restriction digest of PCR products amplified from genomic DNA using primers 5’-TGTCTCATTCGTCATTACCGTC-3′ and 5’-AAGTTTCCGTGAAATTGAGGAG-3′. *gas6* mutants (alleles um296, um297, um298 and um299) were genotyped by XcmI restriction digest of PCR products amplified from genomic DNA using primers 5’-GCGAACACATTGAGCAAGAA-3′ and 5’-CATCG- CTAATGCTTCATCCA-3′.

### Genotyping embryos post ISH and immunostaining

*hoxb1a*, *hnf1ba* and *valentino* homozygous mutants are not viable as adults. As a result, all embryos assayed in this study were collected from crosses of heterozygous parents. After ISH and immunostaining, embryos were thoroughly rinsed in 1xPBS solution and individually genotyped. Representative genotyping data for *hoxb1a* mutants are shown in Additional file 1: Figure S1. Embryos lacking r5 *krox20* staining represent *valentino* and *hnf1ba* homozygous mutants. Mutants ordered from ZIRC were genotyped as described above.

### mRNA injections

All mRNAs for microinjection were synthesized in vitro using the Sp6 mMessage mMachine Kit (Ambion). 100 ng/ul each of GFP [51] and *hoxb1a* [28] mRNA were combined and 1-2 ng of this mixture was injected into early 1-cell stage embryos.

### *olig2* reporter line in *gas6* mutant background

The transgenic line *Tg (olig2:EGFP)*^*vu12*^ was crossed into the *gas6* mutant background and subsequently a *gas6* homozygous mutant line was generated carrying the *olig2:eGFP* transgene. This line was used in preparing the RNA-seq library as well as studying the status of Olig2+ oligodendrocyte progenitor cells in mutant background.

### RNA-seq library preparation

*gas6* mutant embryos carrying the *olig2:EGFP* transgene were raised to 48hpf. Using the GFP signal as a guide, hindbrains were dissected from homozygous *gas6* transgenic mutants. Hindbrains were also dissected from *Tg (olig2:EGFP)*^*vu12*^ embryos as control samples. Pools of dissected tissues were deyolked and total RNA was extracted using the RNeasy Mini Kit (Qiagen). Similarly, WT and *hoxb1b*^*um197*^ mutants were collected at 18hpf and total RNA was extracted from pools of dechorionated, deyolked, whole embryos. For each RNA-seq experiment, three libraries were synthesized from 3μg RNA for each WT and mutant sample using the TruSeq Stranded mRNA Library Prep Kit (Illumina). All libraries were analyzed for quality on a bioanalyzer prior to sequencing (Agilent 2100 BioAnalyzer).

### Processing and analysis of RNA-seq data

Fastq files were analyzed as previously described [55] using the University of Massachusetts Medical School Dolphin web interface [60]. Differentially-expressed (DE) genes were identified as those with a greater than 2-fold change in expression between the WT and mutant samples. RNA-seq data is available at GEO under accession number GSE113437.

### Quantitative PCR

Total RNA was extracted from whole embryos (WT and *hoxb1b*^*−/−*^ at 18hpf), or from dissected hindbrain tissue (*gas6*^*−/−*^ and WT at 48hpf) using the RNeasy Mini Kit (Qiagen). Approximately 100 ng of RNA was used to reverse transcribe cDNA using the High Capacity cDNA Reverse Transcription Kit (Applied Biosystems). The qPCR reaction was carried out using SYBR Green qPCR Master Mix (BioTool) on an Applied Biosystems 7300 PCR System.

## Results

### Derivation of gene-sets expressed in r4 and r5/r6 of the zebrafish hindbrain

To generate a list of candidate genes for function in the formation of r4-r6 of the vertebrate hindbrain, we turned to the gene expression database hosted at ZFIN [61]. We downloaded the “Expression data for wildtype fish” file and searched for genes whose annotation include the terms “hindbrain”, “rhombomere 4”, “rhombomere 5” or “rhombomere 6”. This produced a list of 1820 entries (Additional file 2: Table S1). We eliminated 146 records representing expressed sequence tags (ESTs), as these entries are not fully annotated, resulting in 1674 genes. To further characterize these genes, we next manually reviewed the expression patterns deposited in ZFIN. Since we were particularly interested in genes controlling rhombomere formation, we excluded 480 genes that are only expressed later than 24hpf – when rhombomere formation is already completed – leaving 1194 genes. We also expect genes controlling rhombomere formation to be expressed throughout the corresponding rhombomere. For instance, *hoxb1a* and *valentino*, which are respectively active in r4 and r5/r6 formation [5, 22, 23], are expressed throughout the entire corresponding rhombomere, while *islet1*, which is required for the differentiation of specific neurons, is expressed only in a subset of cells in each rhombomere. After restricting ourselves to genes expressed throughout one, or more, rhombomeres, we were left with 107 genes expressed in r4, r5 or r6 prior to 24hpf. Specifically, 68 of these genes are expressed in r5 and/or r6 (r5/r6 gene-set), while 39 are expressed in r4 (r4 gene-set). Notably, expression of these genes is not necessarily exclusive to r4 or r5/r6, but many of them are also expressed in additional rhombomeres – particularly r3. Our literature review revealed that a relatively small fraction of these 107 genes has previously reported roles in hindbrain formation. Specifically, eleven members of the r4 gene-set (28%) and twelve of the r5/r6 gene-set (18%) have been previously implicated in hindbrain formation, indicating that a large number of uncharacterized genes are expressed in zebrafish r4-r6.

### PG1 *hox* function is not required for expression of many r4 genes

We next set out to position the r4 and r5/r6 gene-sets within the GRN controlling caudal hindbrain formation. Previous work demonstrated that mutations in the PG1 *hox* TFs *hoxb1a* and *hoxb1b* disrupt proper hindbrain formation in zebrafish. In particular, *hoxb1b* mutants possess smaller r4 and r6, while *hoxb1a* mutants display a mis-specified r4 [24–26], suggesting that PG1 *hox* TFs may regulate the r4 GRN.

**Fig. 1:**
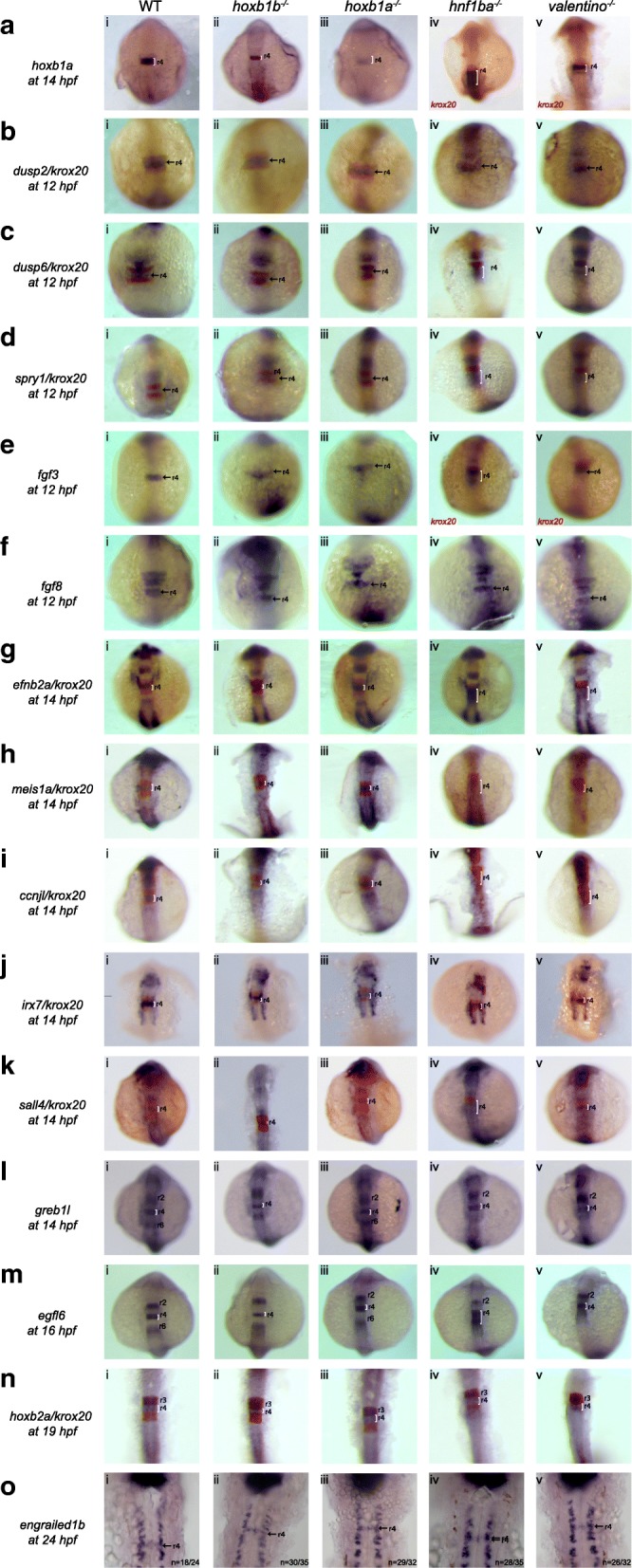
PG1 *hox* function is not required for expression of most r4 genes. Expression of r4 genes was assayed via ISH in (i) WT, (ii) *hoxb1b* mutant, (iii) *hoxb1a* mutant*,* (iv) *hnf1ba* mutant and (v) *valentino* mutant zebrafish. The genes assayed include **a** *hoxb1a,* **b** *dusp2*, **c** *dusp6*, **d** *spry1*, **e** *fgf3*, **f** *fgf8*, **g** *efnb2a*, **h** *meis1a*, **i** *ccnjl*, **j** *irx7*, **k** *sall4*, **l** *greb1l*, **m** *egfl6*, **n** *hoxb2a* and **o** *engrailed1b*. *krox20* (red) which is expressed in r3 and r5, was used to assign the expression domains of several genes, as indicated. All embryos are oriented in dorsal view with anterior to the top. Embryos collected at 12hpf, 14hpf, 16hpf and 19hpf were imaged as whole-mounts. 24hpf embryos were flat-mounted for imaging. Black arrows point to the r4 domain in 12hpf and 24hpf embryos. White brackets mark the normal and expanded r4 domains in embryos staged at 14hpf, 16hpf and 19 hpf

To directly test if genes from the r4 gene-set are key components of a PG1 *hox*-regulated r4 GRN, we analyzed expression of the r4 gene-set by ISH in *hoxb1a* and *hoxb1b* mutant zebrafish (Fig. 1). Specifically, since mutants for two known key r5 regulators *(hnf1ba* and *valentino*) show near-complete loss of r5 gene expression [22, 23], we examined if PG1 *hox* mutants display a similarly profound effect on expression of the r4 gene-set. Zebrafish *hoxb1b* is required for expansion of the r4 domain, but not for r4 formation [24, 26]. Accordingly, homozygous *hoxb1b* ^*um197/um197*^ mutants (hereafter referred to as *hoxb1b* mutants) possess a narrow r4 domain that nevertheless expresses *hoxb1a* and is capable of generating both Mauthner cells and nVII facial neurons [24, 26], albeit at a lower rate than wildtype r4. We generated ISH probes for 14 genes from the r4 gene-set and find that all 14 remain expressed in *hoxb1b* mutant fish, although their expression domains are reduced in size due to the smaller r4 (Fig. 1, column ii). Although there may be subtle changes in expression of some of the r4 genes tested, these are much less pronounced than what is observed in *hnf1ba* and *valentino* mutants ([22, 23]; such differences may instead result from slight variations in the ISH processing), suggesting that *hoxb1b* is not a key regulator of r4 formation. In contrast to *hoxb1b, hoxb1a* is required for r4 formation [24]. In particular, homozygous *hoxb1a*^*um191/um191*^ mutant embryos (hereafter referred to as *hoxb1a* mutants), have reduced *hoxb1a* expression, lack r4-specific Mauthner cells, and the nVII facial neurons fail to migrate out of r4. While this disruption of r4 formation suggests that r4 gene expression might be generally reduced in *hoxb1a* mutants, we instead find that expression of the r4 gene set persists in *hoxb1a* mutants (Fig. 1, column iii), at least until 24hpf (Additional file 3: Figure S2). Previous work [24, 26] showed that expression of several r4 genes is only subtly affected in *hoxb1a/hoxb1b* double mutants relative to single mutants. While this suggests that the PG1 *hox* genes do not display significant functional redundancy, it remains possible that a subset of r4 genes may be redundantly regulated by the two PG1 *hox genes*.

Our finding that PG1 *hox* function is not required for expression of the 14 tested r4 genes led us to assess more broadly if hindbrain gene expression is *hox*-dependent. To address this, we took advantage of the viability of homozygous *hoxb1b* mutants and used RNA-seq to identify *hoxb1b*-dependent genes during zebrafish embryogenesis. Comparing the *hoxb1b* mutant transcriptome to that of wildtype embryos revealed 866 differentially expressed genes at 18hpf (Additional file 4: Figure S3) (Additional file 5: Table S2). Comparison to the hindbrain-expressed genes identified in our database search demonstrated that only 85 of these genes are affected in *hoxb1b* mutants (seven up-regulated and 78 down-regulated; Additional file 4: Figure S3B). Thus, by this measure, ~ 5% (85/1674) of zebrafish hindbrain genes are *hoxb1b* regulated (although the fraction may be lower, since some of these genes are also expressed in non-hindbrain tissue). Furthermore, of the 85 genes, only four (*mpz, fgf8a, cyp26b1* and *desma*) have rhombomere-restricted expression patterns (Additional file 5: Table S2). Also, while *fgf8* and *mpz* were identified as upregulated in *hoxb1b* mutants by our RNA-seq analysis*,* we did not detect obvious changes in expression of these genes by ISH (Figs. 1Fii and 3Cii). Since RT-qPCR on independently collected samples validated our RNA-seq analysis (Additional file 4: Figure S3D), it is possible that gene expression changes identified by RNA-seq are too subtle for detection by ISH. Indeed, the change in expression of *fgf8* and *mpz* is less than 2.5-fold and we find that the majority of *hoxb1b*-regulated genes identified by RNA-seq show relatively subtle changes in expression, such that ~ 93% of the down-regulated genes are reduced by less than 4-fold and only three genes are down-regulated by more than 10-fold (Additional file 5: Table S2). We conclude that, while many genes may be *hoxb1b*-regulated in the zebrafish embryo, only a few of these genes are expressed in the hindbrain and the observed changes in expression levels are relatively subtle. Hence, our ISH analysis in *hoxb1a* and *hoxb1b* mutants, together with our RNA-seq analysis of *hoxb1b* mutants, suggests that PG1 *hox* genes may not be absolutely required for r4 gene expression. Further, a recent RNA-seq analysis of *Hoxa1* mutant mouse embryos (murine *Hoxa1* is functionally analogous to zebrafish *hoxb1b*) identified 1537 *Hoxa1*-dependent genes [48], but only 31 genes are shared between the zebrafish and mouse data sets (Additional file 6: Figure S4), suggesting that PG1 *hox* genes may in fact regulate distinct sets of genes in different species.

### A subset of r4 genes is regulated by FGF, but not RA, signaling

**Fig. 2:**
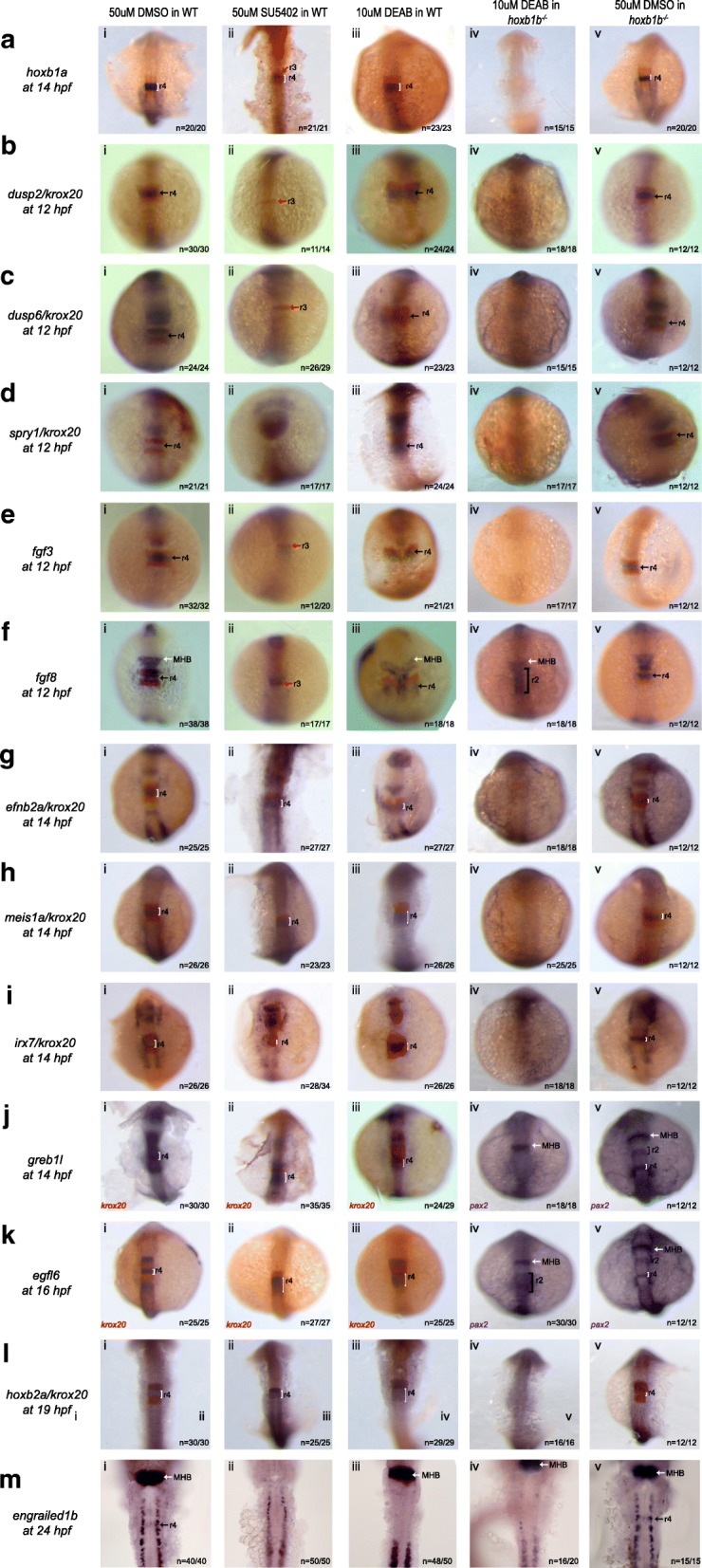
Simultaneous loss of *hoxb1b* and RA function disrupts expression of r4 genes. Expression of r4 genes was assayed via ISH in (i) WT embryos treated with 50uM DMSO, (ii) WT embryos treated with 50uM SU5402 (iii) WT embryos treated with 10uM DEAB, (iv) *hoxb1b* mutant embryos treated with 10uM DEAB and (v) *hoxb1b* mutant embryos treated with 10uM DMSO. The genes assayed include **a** *hoxb1a,* **b** *dusp2*, **c** *dusp6*, **d** *spry1*, **e** *fgf3*, **f** *fgf8*, **g** *efnb2a*, **h** *meis1a*, **i** *irx7*, **j** *greb1l*, **k** *egfl6*, **l** *hoxb2a* and **m** *engrailed1b*. *krox20* (red), which is expressed in r3 and r5, was used to position the expression domains of several genes, as indicated. In panels (Jiv), (Jv), (Kiv) and (Kv), *pax2* is the second blue marker which labels the MHB. Black arrows point to r4, red arrows to r3 and white arrows to the MHB. White and black brackets indicate r4 and r2 size, respectively. All embryos are oriented in dorsal view with anterior to the top. Embryos collected at 12hpf, 14hpf, 16hpf and 19 hpf were imaged as whole-mounts. 24hpf embryos were flat-mounted for imaging. This figure also shows that a subset of r4 genes is regulated by FGF signaling (Bii, Cii, Dii, Eii and Fii) and that r4 genes are not affected by the loss of RA signaling (embryos in row iii)

The possibility that PG1 *hox* genes are not required for r4 gene expression suggests that other factors may be involved. In particular, the RA and FGF signaling pathways are known to function in hindbrain development [8–13]. To determine if expression of the r4 gene-set is dependent on RA or FGF signaling, we treated wildtype embryos with 50uM SU5402 (a competitive inhibitor of the FGF receptor tyrosine kinase; [19]) or 10uM DEAB (a competitive inhibitor of RALDH, the enzyme required for conversion of retinaldehyde to retinoic acid; [10, 24]). We find that inhibition of FGF signaling blocks expression of *dusp2* (Fig. 2Bii), *dusp6* (Fig. 2Cii), *spry1* (Fig. 2Dii), *fgf3* (Fig. 2Eii), and *fgf8* (Fig. 2Fii), in r4, although the effect on *spry1* is difficult to assess since the anterior *spry1* expression domain appears to have expanded. In contrast, inhibition of Fgf signaling does not block expression of *hoxb1a* or the remaining members of the r4 gene-set, with the exception of *engrailed1b* (Fig. 2)*.* Notably, all genes affected by loss of FGF signaling are themselves involved in the FGF signaling pathway, confirming the extensive use of feedback loops in this pathway [62–64]. Furthermore, inhibiting RA signaling does not block expression of the r4 genes tested, again with the exception of *engrailed1b* (Fig. 2Miii). The fact that expression of the r4 gene-set is not lost upon disrupting PG1 Hox TFs, FGF signaling or RA signaling suggests that it is either regulated independently of these signaling pathways or is under combinatorial control.

### Simultaneous loss of *hoxb1b* and RA function disrupts expression of r4 genes

We previously found that *hoxb1a* expression is unaffected when RA and *hoxb1b* function is disrupted independently, but is lost when these signals are disrupted simultaneously [24]. To determine if the r4 gene-set is similarly regulated, we treated *hoxb1b* mutant embryos with 10uM DEAB and assayed gene expression by ISH. We find that expression of ten members of the r4 gene-set is completely lost when *hoxb1b* and RA signaling are simultaneously disrupted (Fig. 2, column iv). Two genes, *fgf8* (Fig. 2Fiv), and *egfl6* (Fig. 2Kiv), show residual expression in the hindbrain, but these two genes are normally expressed also in the anterior hindbrain and previously published mouse data showed an expansion of the r2/r3 domains upon disruption of RA signaling [65]. Hence, the residual *fgf8* and *egfl6* expression detected in DEAB-treated *hoxb1b* mutants may be derived from r2/r3, not from r4. However, expression of *greb1l* (Fig. 2Jiv) – which is also expressed in the anterior hindbrain – is completely lost, indicating that not all genes are regulated in the same manner. A recent study reported subtle changes in *fgf3/8a* expression patterns in *hoxb1b* mutants and suggested that *hoxb1b* may regulate FGF signaling [26]. In line with this observation, we show that expression of FGF pathway components (*fgf3, fgf8, dusp2, dusp6* and *spry1*) is lost upon simultaneous disruption of *hoxb1b* and RA function, indicating that FGF signaling is downstream of *hoxb1b* and RA activity in the hindbrain. Notably, simultaneous loss of *hoxb1b* and RA function has no effect on *pax2* (Fig. 2Jiv and Kiv) and *fgf8* (Fig. 2Fiv) expression at the mid-hindbrain boundary (MHB), indicating that co-regulation is specific to the region where *hoxb1b* is expressed. We carried out an analogous experiment to test if *hoxb1b* and FGF also cooperate to control r4 gene expression but find that even brief treatment of *hoxb1b* mutants with SU5402 dramatically disrupts embryogenesis (Additional file 7: Figure S5), precluding us from assaying hindbrain gene expression. We conclude that expression of the r4 gene-set (including FGF pathway components) requires both *hoxb1b* and RA function.

### Expression of the r5/r6 gene-set is dependent on *hnf1ba* and *valentino* function

**Fig. 3:**
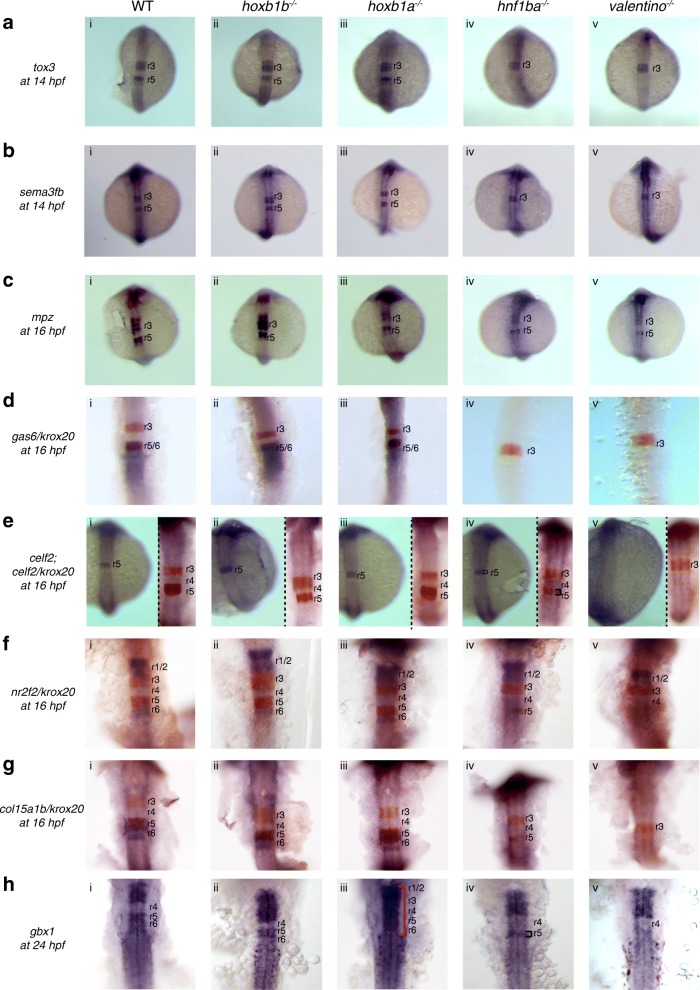
Expression of the r5/r6 gene-set is dependent on *hnf1ba* and *valentino*. Expression of r5/r6 candidate genes was assayed via ISH in (i) WT, (ii) *hoxb1b* mutant, (iii) *hoxb1a* mutant*,* (iv) *hnf1ba* mutant and (v) *valentino* mutant zebrafish lines. The genes assayed include **a** *tox3*, **b** *sema3fb*, **c** *mpz*, **d** *gas6*, **e** *celf2*, **f** *nr2f2*, **g** *col15a1b* and **h** *gbx1*. *krox20* (red), which is expressed in r3 and r5, was used to position the expression domains of several genes as indicated. All embryos are oriented in dorsal view with anterior to the top. Embryos collected at 12hpf, 14hpf, 16hpf and 19hpf were imaged as whole-mounts. 24hpf embryos were flat-mounted for imaging. Black brackets mark the smaller r5 domain. Red bracket in (Hiii) indicates expression of *gbx1* throughout the hindbrain, as a result of reappearance of *gbx1* expression in r4 of *hoxb1a* mutants

*hnf1ba* is the earliest-acting TF in zebrafish r5/r6 where it controls expression of the *mafB* gene *valentino.* Indeed, previous work demonstrated that both *hnf1ba* and *valentino* function is required for the expression of several r5/r6 genes [23]. In order to determine if expression of our r5/r6 gene-set is also dependent on *hnf1ba* and *valentino*, we generated ISH probes for eight genes (*gbx1*, *tox3, sema3fb, mpz, gas6, celf2, nr2f2* and *col15a1b*) and assessed their expression in homozygous *hnf1ba*^*hi2169/hi2169*^ (referred to as *hnf1ba* mutant) and homozygous *valentino*^*b337/ b337*^ (referred to as *valentino* mutant) embryos. For each of the eight genes, we find that expression is dramatically reduced in r5 and r6 of both mutant lines (Fig. 3, columns iv and v). Expression of *tox3, sema3fb, gas6, nr2f2* and *col15a1b* appears to be completely lost in r5/r6 of both mutant lines, whereas residual expression of *celf2* (Fig. 3Eiv) *and gbx1* (Fig. 3Hiv) is detected in *hnf1ba* mutants and weak *mpz* (Fig. 3Civ, v) expression is observed in both *hnf1ba* and *valentino* mutants. This is in agreement with previous reports [66] demonstrating that slight expression of some r5 genes may persist in these mutants.

*hoxb1b* is initially broadly expressed in the caudal hindbrain [10, 16] and previous work demonstrated that several r5/r6 genes (including seven genes from the r5/r6 gene set; Additional file 1: Table S1) are up-regulated following *hoxb1b* overexpression [51], suggesting that *hoxb1b* may regulate gene expression in r5/r6. We therefore assayed expression of the r5/r6 gene-set also in *hoxb1b* and *hoxb1a* mutant embryos (Fig. 3, column ii and iii), but find that r5/r6 gene expression persists in PG1 *hox* mutants. We conclude that *hnf1ba* and *valentino*, but not *hoxb1b* or *hoxb1a*, are required for gene expression in r5 and r6.

### Expression of the r5/r6 gene-set requires FGF and RA signaling

**Fig. 4:**
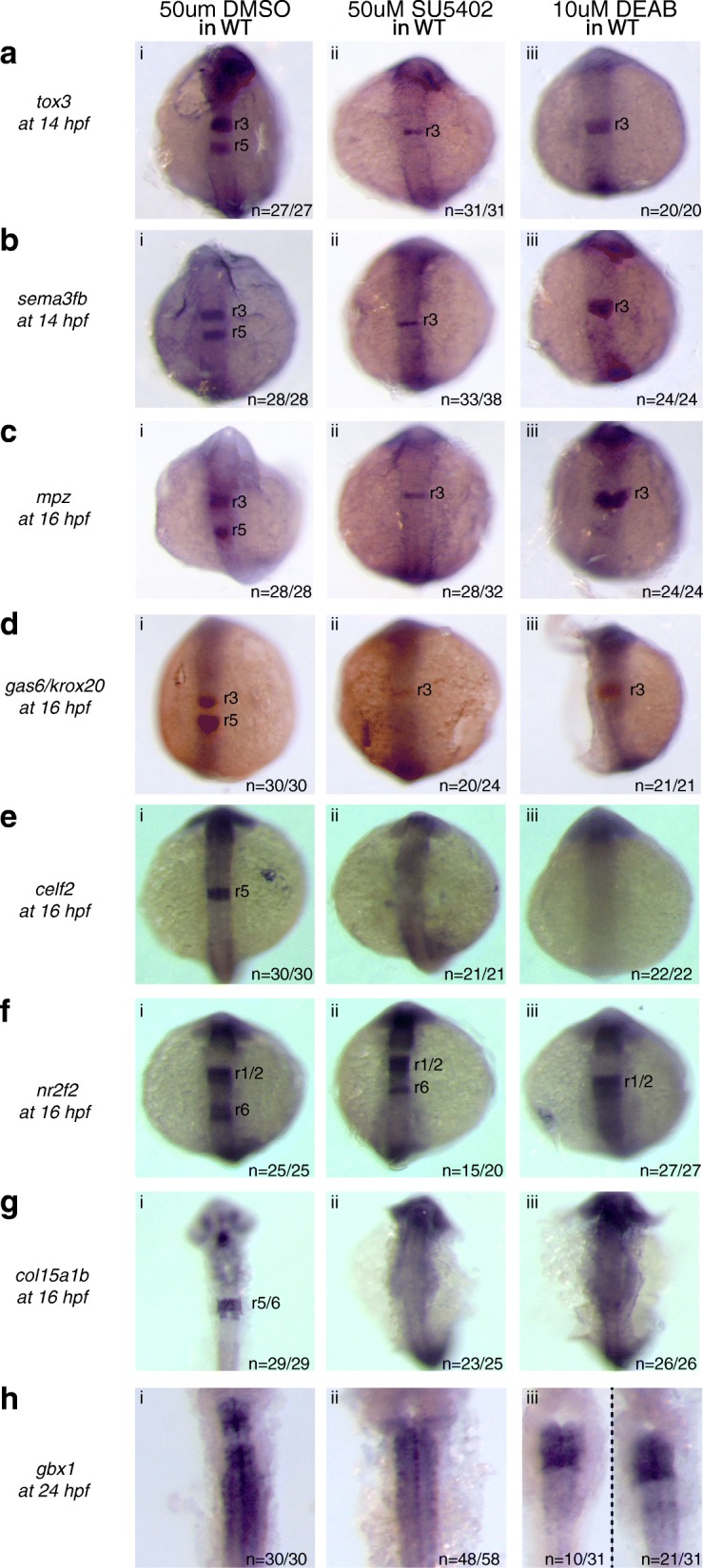
Expression of the r5/r6 gene-set requires FGF and RA signaling. Expression of r5/r6 genes was assayed via ISH in (i) WT embryos treated with 50uM DMSO, (ii) WT embryos treated with 50uM SU5402 *and* (iii) WT embryos treated with 10uM DEAB. The genes assayed include **a** *tox3*, **b** *sema3fb*, **c** *mpz*, **d** *gas6*, **e** *celf2*, **f** *nr2f2*, **g** *col15a1b* and **h** *gbx1*. *krox20* (red), which is expressed in r3 and r5, was used to position the expression domain of some genes as indicated. All embryos are oriented in dorsal view with anterior to the top. Embryos collected at 12hpf, 14hpf, 16hpf and 19hpf were imaged as whole-mounts. 24hpf embryos were flat-mounted for imaging

Previous work demonstrated that FGF signaling is required for r5/r6 formation [19, 20]. Since *hnf1ba* expression is independent of FGF [20], FGF must control r5/r6 formation downstream of this transcription factor. Indeed, FGF reportedly acts together with *hnf1ba* to regulate *valentino* and *krox20* expression [23, 30]. Hence, by determining if the r5/r6 gene-set is FGF independent (like *hnf1ba*) or FGF dependent (like *valentino* and *krox20*), we can better understand the GRN controlling r5/r6 formation. Additionally, RA is required for formation of r5/r6 and for the expression of r5/r6-restricted genes such as *hnf1ba*, *valentino*, and *krox20* [16]. Strikingly, our analyses revealed that inhibition of either FGF or RA signaling in wildtype embryos blocks expression of all genes in the r5/r6 gene-set (Fig. 4), with the one exception of a narrow domain of residual *nr2f2* expression in r6 (Fig. 4Fii) of SU5402 treated embryos. Hence, r5/r6 gene expression is dependent both on the activity of the *hnf1ba* and *valentino* TFs, as well as on RA and FGF signaling.

### *hnf1ba* establishes the posterior boundary of r4 gene expression

Gene expression boundaries in the developing hindbrain are initially established via repressive interactions at the level of transcription. For instance, *hoxb1a, efnb2a* and *fgf3* expression expands caudally from r4 into presumptive r5 in *hnf1ba* mutants [23, 36], indicating that *hnf1ba* represses r4 gene expression (directly or indirectly) to establish the r4/r5 border. In order to determine if the expression domains of genes in the r4 and r5/r6 gene-sets are similarly established by repressive interactions, we examined r4 gene expression in *hnf1ba* and *valentino* mutants, as well as r5/r6 gene expression in the PG1 *hox* mutants.

For the r4 gene-set, we find that *dusp6* (Fig. 1Civ), *spry1* (Fig. 1Div), and *egfl6* (Fig. 1Miv), show expansion of the r4 expression domain into r5 of *hnf1ba* mutants, while *fgf8, irx7, greb1l* and *eng1b* expression is not affected. In contrast, expression of the r4 gene-set is not affected in *valentino* mutants, with the exception of *efnb2a* (Fig. 1Gv)*,* which may show a slight expansion into r5 (previously shown in [23]). For the r5/r6 gene-set, we do not observe expansion into r4 in either of the PG1 *hox* mutants (Fig. 3, columns ii and iii). We conclude that *hnf1ba* restricts expression of many, but not all, genes in the r4 gene-set to presumptive r4, but that *valentino* and the PG1 *hox* genes are not required to establish gene expression boundaries for the r4 and r5/r6 gene-sets.

### *gbx1* expression requires *hnf1ba* and *valentino* in r5/r6 and is repressed by *hoxb1a* in r4

**Fig. 5:**
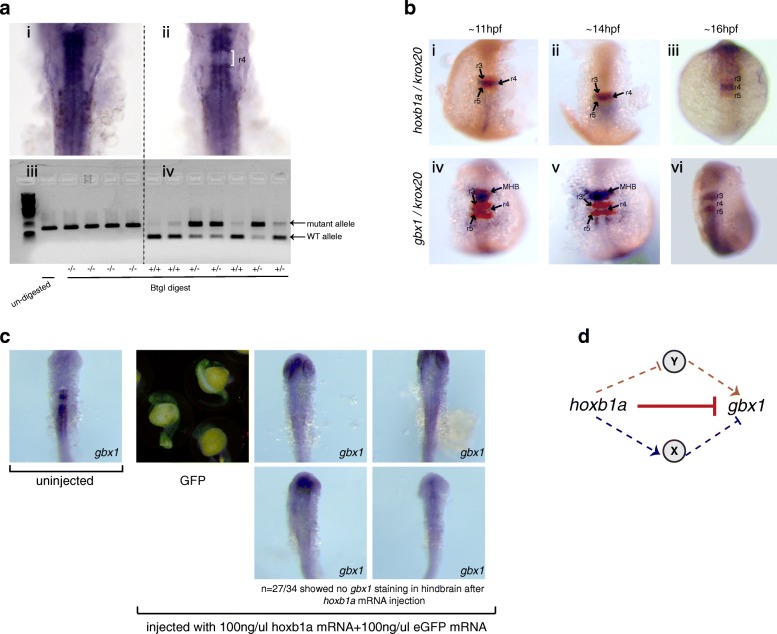
*gbx1* expression is repressed by *hoxb1a* in r4. **a** *gbx1* expression is restored in *hoxb1a* mutant embryos. Embryos from a cross of *hoxb1a* heterozygous parents were assayed by ISH for *gbx1* expression, producing two phenotypes (i, ii). Subsequent genotyping revealed that homozygous *hoxb1a* mutants express *gbx1* in r4 (ii, iv). **b** *gbx1* is initially expressed in r4 (iv), but disappears (v, vi) when *hoxb1a* expression is activated (i, ii, iii). **c** A mixture of *hoxb1a* and GFP mRNA was injected into 1-cell stage embryos and successfully injected embryos (identified by GFP expression) were stained for *gbx1* expression at 24hpf. The observed reduction in *gbx1* expression demonstrates that *hoxb1a* is capable of repressing *gbx1* expression. **d** Hypothetical model depicting the potential relationship between *gbx1* and *hoxb1a*. *hoxb1a* could either repress *gbx1* directly (solid red T bar) or indirectly by activating a repressor (X; blue arrow) or repressing an activator (Y; orange T bar)

The *gbx1* gene displays an interesting expression pattern in that it is expressed throughout the hindbrain at 24hpf, except in r4 (Fig. 3Hi). To better understand the regulation of *gbx1* expression, we analyzed its expression pattern in *hoxb1b, hoxb1a, hnf1ba* and *valentino* mutants. We find that *gbx1* expression is lost in r5/r6 of *valentino* mutants and that only a narrow expression domain persists in r5 of *hnf1ba* mutants (Fig. 3Hiv, v), indicating that *hnf1ba* and *valentino* are required for *gbx1* expression in r5/r6. Strikingly, *gbx1* expression is restored to the r4 domain of 24hpf *hoxb1a* mutant embryos, but not of *hoxb1b* mutant embryos (Figs. 3Hiii and 5Ai), suggesting that *gbx1* might be repressed by *hoxb1a* in r4. *gbx1* is actually expressed in wildtype r4 at ~10hpf, but this expression disappears coincident with the onset of *hoxb1a* expression (Fig. 5b), again suggesting that *hoxb1a* may repress *gbx1* expression. To test this directly, we overexpressed *hoxb1a* by injection of synthetic mRNA into 1–2 cell stage embryos and assayed *gbx1* expression at 24hpf by ISH. We find that ~ 80% (27/34) injected embryos display a clear decrease in *gbx1* hindbrain expression (Fig. 5c). We conclude that *hoxb1a*, either directly or indirectly, represses *gbx1* expression in r4 (Fig. 5d) and that its expression in r5/r6 is regulated similarly to the genes in the r5/r6 gene-set.

### *gas6, gbx1, sall4, egfl6, celf2* and *greb1l* function is not required for r4-r6 formation

**Fig. 6:**
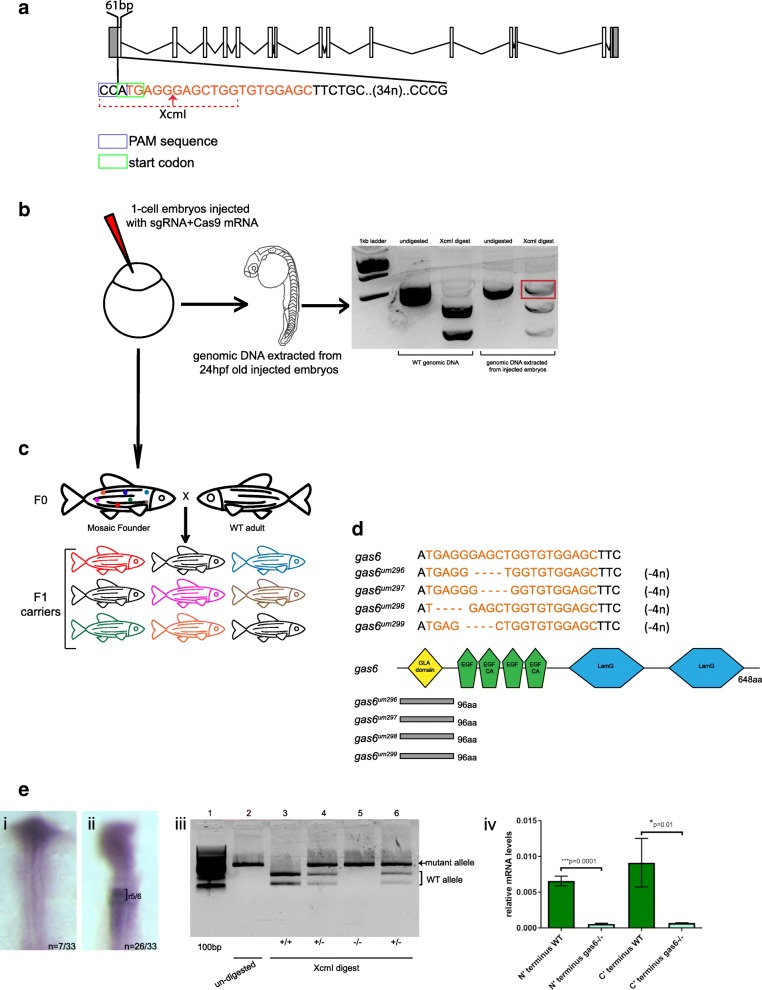
Scheme for generating *gas6* mutant line. **a** Schematic showing the 20 nucleotide (orange text) target site in exon 1 of *gas6*. CAA represents the PAM sequence (blue box) and ATG (green box) is the start codon. XcmI target sequence is indicated by the dotted red line, the red arrow denotes the cut site. **b** sgRNA and Cas9 mRNA was injected into 1-cell stage embryos. Injected embryos were raised to 24hpf and genomic DNA was extracted from a pool of embryos. XcmI digest of PCR products amplified from genomic DNA (extracted from injected embryos) reveal the presence of a mutation (red box in gel). **c** Injected embryos were raised to give rise to F0 adults. These fish were crossed with WT adults to raise the F1 generation. At 3 months age, genomic DNA was extracted from fin-clips from individual F1 fish and genotyped as in panel B. **d** Sequencing of F1 genomic DNA revealed transmission of four different mutant alleles (um296, um297, um298, um299), each with a different 4 nucleotide deletions (orange dashes). Each mutant allele codes for 96 out of frame amino acids (gray boxes) followed by a premature stop codon. **e** One quarter of the embryos collected from a cross of two heterozygous parents lack *gas6* expression in r5/r6 (i). XcmI digest of PCR products amplified from genomic DNA extracted from embryos lacking *gas6* expression were homozygous for mutant *gas6* allele (iii, lane 5). cDNA was synthesized from total RNA extracted from WT and homozygous *gas6* mutant fish. Quantitative RT-PCR using two different primer pairs (targeting the N and C termini, respectively) shows that homozygous *gas6* mutants have significantly lower levels of *gas6* mRNA (iv)

Considering the number of genes assigned to the r4 and r5/r6 gene-sets by our database search, it is surprising that previous large-scale mutagenic screens identified only a few genes required for r4-r6 formation [22, 33, 37]. While this finding may indicate redundancy in the r4 and r5/r6 GRNs, the fact that targeted mutagenesis studies have identified additional genes required for r4-r6 formation (e.g. PG1 *hox* genes [24–26, 67–69] and *krox20* [38, 70]) may instead suggest that the original screens did not reached saturation. To directly test if genes in the r4 and r5/r6 gene-sets are required for rhombomere formation, we selected six genes (*gas6, sall4, egfl6, celf2, greb1l* and *gbx1*) for further analysis. Germline mutations for four of these genes (*sall4, egfl6, celf2 and greb1l*) have been generated by community-based mutagenesis projects [59, 71] and are available from the zebrafish resource center (ZIRC; Table 1). These four mutations were procured from ZIRC in the form of fertilized embryos and raised in our laboratory. Genotyping and sequencing confirmed the presence of the expected mutations (Table 1) (Additional file 8: Figure S6). Since *gas6* and *gbx1* mutants were not available from the resource center, we generated these by CRISPR/Cas9-mediated mutagenesis. We designed a sgRNA targeting the *gas6* start codon in exon 1 and identified two *gas6* mutant founders from five fish screened (Fig. 6a-c). One founder did not transmit mutation to its offspring (*n* = 0/92), but the other transmitted mutations to 20% (*n* = 12/60; Table 2) of its F1 offspring. Sequencing revealed that this founder transmitted four different mutant alleles (Additional file 9: Data S1) where each allele carried a different four nucleotide deletion, but translation of each mutant allele is nevertheless predicted to produce an out of frame product that terminates at the same premature stop codon (residue 99; Fig. 6d) (Additional file 9: Data S1). ISH and RT-qPCR analyses further revealed that *gas6* transcripts are lacking in *gas6* mutants, possibly as a result of nonsense-mediated mRNA decay (Fig. 6e). In an analogous fashion, a sgRNA was designed to exon 1 of *gbx1* (Additional file 10: Figure S7A) and we identified two founders (Additional file 10: Figure S7B) (Table 2). Of the two founders, only one produced offspring and this founder transmitted two different mutations. Both of these alleles produce premature STOP codons N-terminal to the homeodomain (Additional file 10: Figure S7D) (Additional file 11: Data S2).

**Table 1:** Mutant lines obtained from ZIRC

Gene name	Mutant ID	Chromosome location	Exon affected	Mutation	Consequence	Amino acid affected
*greb1l*	sa17608	chr2:11980696	1 of 32	T > A	nonsense	41 of 1942aa
*celf2*	sa33469	chr4: 17566280	2 of 13	A > T	nonsense	28 of 514aa
*egfl6*	sa21615	chr9: 54710599	8 of 12	G > A	disrupted splice site	277 of 506aa
*sall4*	sa14110	chr23: 39233081	2 of 4	C > T	nonsense	695 of 1091aa

**Table 2:** Summary of transmission and viability of mutant lines

Gene name	F0 generation	F1 generation	F2 generation
*gas6*	5 fish screened, 2 founders	Founder 1: 12/60 carried mutations; transmitted 4 different alleles, each resulting in a frame-shift mutation Founder 2: 0/92 carried mutations	5/16 homozygous mutant
*gbx1*	2 fish screened, 2 founders,	Founder 1: 34/48 carried mutations; transmitted 4 different alleles – 2 resulted in frame-shift mutations, 2 did not Founder 2: did not produce offspring	0/15 homozygous mutant
*sall4*	N/A	^a^8/24	5/21 homozygous mutant
*greb1l*	N/A	^a^10/24	5/34 homozygous mutant
*celf2*	N/A	^a^12/24	^b^1/21 homozygous mutant
*egfl6*	N/A	^a^16/24	^c^6/24 homozygous mutant

^a^ZIRC provided offspring of a F1 heterozygous carrier and a WT fish; thus, 50% should be heterozygous carriers

^b^1 homozygous fish identified, it was crossed with a heterozygous sibling for all in situ analyses

^c^homozygous mutants do not breed, all in situ analyses were thus done on crosses of heterozygous carriers

**Fig. 7:**
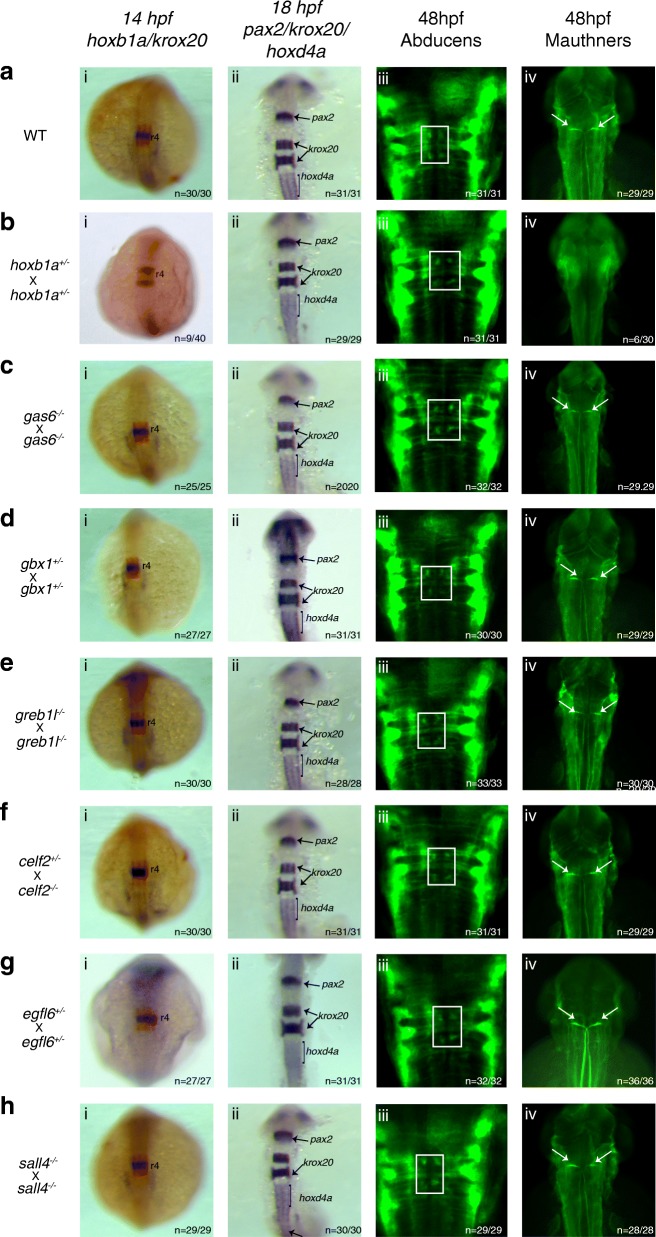
*gas6, gbx1, sall4, egfl6, celf2* and *greb1l* function is not required for r4-r6 formation. ISH for hindbrain markers (i) *hoxb1a* (blue, r4) and *krox20* (red r3/5), (ii) *pax2* (MHB), *krox2*0 (r3/5) and *hoxd4a* (r7-anterior spinal cord), and immunostaining for neuronal markers detecting (iii) abducens motor neurons (four green dots in white boxes) in r5/r6 and (iv) Mauthner neurons (white arrows) in r4 was carried out on embryos collected from an **a** cross of WT fish, **b** cross of *hoxb1a* heterozygous mutants, **c** cross of *gas6* homozygous mutants, **d** cross of *gbx1* heterozygous mutants, **e** cross of *greb1l* homozygous mutants, **f** cross of a *celf2* heterozygous and a homozygous mutant, **g** cross of *egfl6* heterozygous mutants and **h** cross of *sall4* homozygous mutants. All embryos are oriented in dorsal view with anterior to the top. Embryos collected at 14hpf and 18hpf were imaged as whole-mounts. 48hpf embryos were flat-mounted for imaging

In order to assess whether *gas6, gbx1, sall4, egfl6, celf2* and *greb1l* are required for rhombomere formation, we assayed expression of *hoxb1a* in r4, *krox20* in r3 and r5, *pax2* at the MHB boundary and in the otic vesicle, as well as of *hoxd4a* in r7 and anterior spinal cord, by ISH in each of the mutant lines. Upon breeding the mutant lines, we noted that the *gas6, sall4, greb1l and egfl6* lines produced homozygous mutants at close to the expected ratio, while the *celf2* and *gbx1* lines produced few, or no, homozygous mutant offspring (Table 2). This is in agreement with previously published information were *gbx1* mutants obtained via ENU mutagenesis, were also not viable as homozygotes [72]. We also find that homozygous *gas6, sall4,* and *greb1l* mutants are fertile, but homozygous *egfl6* mutants are not. Therefore, our functional analyses made use of offspring from crosses of homozygous mutant parents for *gas6, sall4* and *greb1l,* but offspring of heterozygous carriers for the other lines. Strikingly, we do not detect gene expression changes in any of the mutants (Fig. 7). We also do not detect any defects in rhombomere size, in the spacing of the rhombomere expression domains, or in the integrity of rhombomere boundaries. As a further test of rhombomere development, we examined the differentiation of rhombomere-specific neurons. Specifically, we used immunochemistry to visualize reticulospinal Mauthner neurons in r4 (Fig. 7, column iv) and nVI abducens neurons in r5/r6 (Fig. 7, column iii). Results from this analysis revealed the presence of normal and properly patterned neurons in each of the mutants. Hence, our results suggest that the *gas6, gbx1, greb1l, celf2, egfl6*, and *sall4* genes may not be required for rhombomere-restricted gene expression or neuronal differentiation in the zebrafish hindbrain, although – since we did not assay all aspects of hindbrain development – we cannot exclude the possibility that these genes have other roles in hindbrain development.

### A detailed analysis of *gas6* mutants does not reveal hindbrain defects

**Fig. 8:**
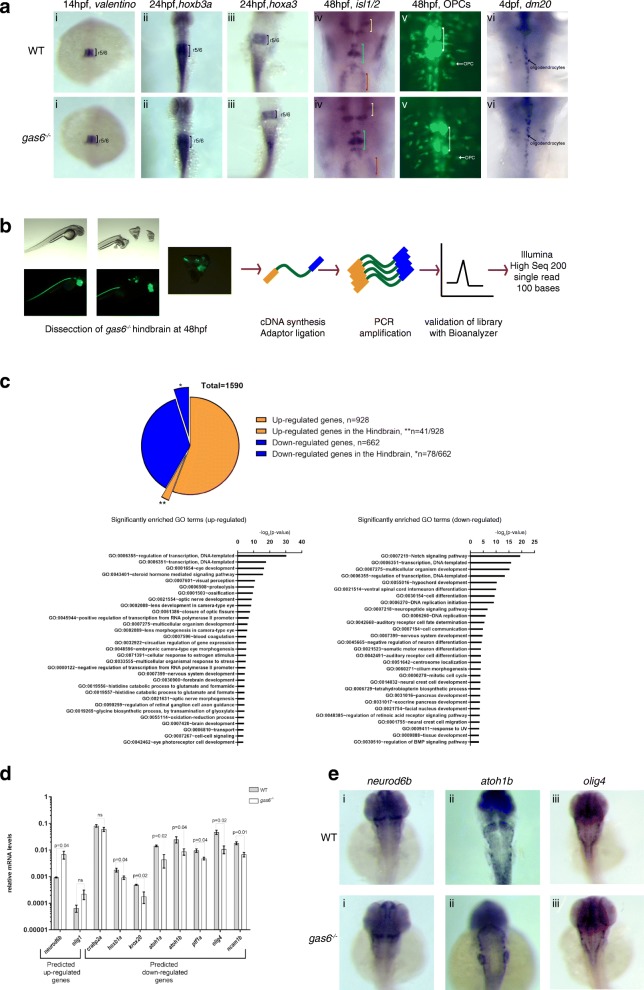
*gas6* may only have subtle roles in caudal hindbrain development. **a** WT and *gas6* mutant embryos were assayed for expression of *valentino* (r5/r6) (i), *hoxb3a* (r5-spinal cord) (ii), *hoxa3* (r5/r6) (iii), *islet1* (cranial nerves)(iv) and *dm20* (oligodendrocyte marker) (vi) by ISH, as well as for the presence of OPCs and abducens neurons by crossing to the *Tg (olig2:EGFP)*^*vu12*^ line (v). In column (iv), yellow brackets mark cranial nerve V, blue brackets mark cranial nerve VII and red brackets mark cranial nerve X. White brackets indicate the presence of abducens (cranial nerve VI) in column (v). **b** Schemes showing RNA-seq library synthesis. Hindbrain tissue was dissected from 48 hpf *gas6* mutant embryos in the *olig2:eGFP* background. Total RNA was collected from pools of hindbrain tissue and was used in library synthesis following the TruSeq Stranded mRNA Library Prep Kit (Illumina) protocol. **c** 1590 differentially expressed genes were identified from RNA-Seq where 41 out of the 928 up-regulated genes and 78 out of the 662 down-regulated genes were expressed in the hindbrain. GO terms related to Biological Processes were identified in both up-regulated and down-regulated genes using DAVID. **d** A subset of differentially expressed genes was validated via qPCR from independently collected hindbrain tissue samples. **e** ISH analysis of representative differentially expressed hindbrain genes (i) *neurod6b*, (ii) *atoh1b* and (iii) *olig4* show no detectable change in expression pattern in *gas6* mutant embryos

To examine the possibility that the mutant lines may have subtle phenotypes that went undetected by our initial screening, we selected the *gas6* mutant for in-depth analysis. Since *krox20* and *valentino* expression is unaffected in *gas6* mutants (Fig. 7Ci, ii and Fig. 8Ai), we reasoned that *gas6* might act downstream of these TFs and therefore examined expression of two later-acting r5/r6 genes (*hoxb3a* and *hoxa3*). However, we find that expression of both genes persists in *gas6* mutants (Fig. 8Ai and ii). We also examined the migration of nVII facial motor neurons from r4 into r5/r6 (Fig. 8Aiv, blue bracket), but do not detect any disruptions of this process in *gas6* mutants. Lastly, r5 and r6 are the source of the initial wave of oligodendrocyte precursor cells (OPCs) (Fig. 8Av) in the hindbrain and we therefore examined expression of *olig2* (a gene required for OPC formation) and *dm20* (a marker of differentiated, myelin-producing oligodendrocytes) (Fig. 8Avi) in *gas6* mutants, but do not find oligodendrocyte formation to be affected in *gas6* mutants*.*

For a more global view of potential defects in *gas6* mutants, we used RNA-seq to compare gene expression between homozygous *gas6* mutants and wildtype embryos. Since *gas6* is expressed exclusively in the hindbrain, we made use of dissected hindbrains from wildtype and *gas6* mutants at 48 hpf (Fig. 8b). Our analysis identified 1590 genes with a 2-fold or greater change in expression between wildtype and mutant hindbrains (928 up-regulated and 662 down-regulated in *gas6* mutants) (Additional file 12: Table S3) (Fig. 8c). Subsequent RT-qPCR analysis on ten differentially expressed genes (*olig1, neurod6b, crabp2a, hoxb1a, krox20, atoh1a, atoh1b, ptf1a, olig4* and *ncam1b*) confirmed the gene expression changes observed by RNA-seq (Fig. 8d). Using the DAVID functional annotation tool [73], we find enrichment for genes associated with developmental processes like “nervous system development”, “forebrain development”, and “neural crest development”, but only a few genes are associated with each GO term. Furthermore, comparison to the hindbrain-expressed genes identified in our database search (Additional file 2: Table S1) revealed that only ~ 7.5% of the genes differentially expressed in *gas6* mutants (41 up-regulated and 78 down-regulated; Fig. 8c) (Additional file 12: Table S3) are expressed in the hindbrain. However, when we use ISH to assess expression one upregulated (*neurod6b*) and two downregulated *(atoh1b* and *olig4*) genes, we do not detect any differences between wildtype and *gas6* mutant embryos (Fig. 8e). We conclude that disruption of *gas6* leads to changes in hindbrain gene expression, but these changes are too subtle to be detected by ISH and do not seem to affect rhombomere formation or neuronal patterning.

## Discussion

**Fig. 9:**
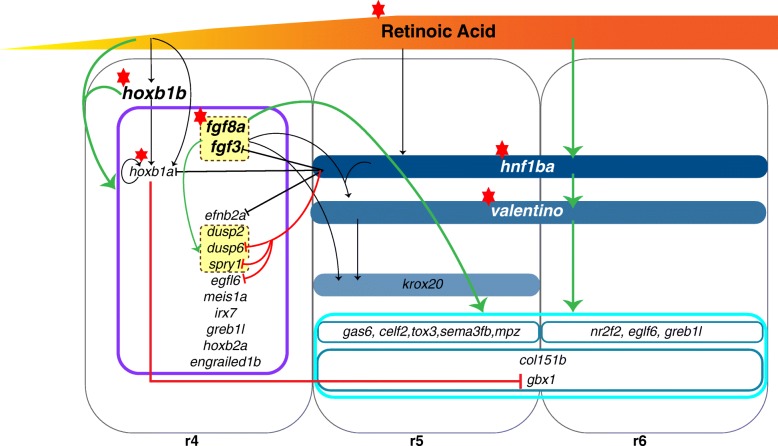
Proposed model depicting the GRN in the caudal hindbrain. Black arrows and bars represent regulatory relationships known prior to this study. Green arrows and red T bars represent relationships uncovered in this study. In this model, arrows (activating) and T bars (repressive) indicate interactions that have been observed, they do not indicate whether the interactions are direct or indirect. All r4 genes regulated by *hoxb1b* + RA are grouped in the purple box. Within r4, *dusp2, dusp6* and *spry1* are regulated by FGF signaling (yellow box). *hnf1ba* represses *dusp6*, *spry1* and *egfl6* expression while *gbx1* expression is repressed in r4 by *hoxb1a*. All r5/r6 genes (light blue box) are regulated by RA, FGF, *hnf1ba* and *valentino.* Red star next to RA, FGFs, *hoxb1b, hoxb1a, hnf1ba* and *valentino* represent the key regulators of the caudal hindbrain – without these factors r4-r6 does not form properly

Our goal for this study was to identify novel genes required for caudal hindbrain development and to position them within the corresponding GRN (Fig. 9). We used the ZFIN database to identify 84 genes that are expressed in r4-r6, but that are relatively uncharacterized. We focused on 22 representative genes and find important differences between r4 and r5/r6 gene expression. In particular, we find that r4 genes are under the combinatorial regulation of RA and *hoxb1b* while r5/r6 genes are under control of RA, FGF, *hnf1ba* and *valentino* in a regulatory arrangement where the loss of any one of these factors disrupts r5/r6 gene expression. Additionally, we identified several novel interactions between the r4 and r5/r6 gene-sets. This includes the repression of *dusp6, spry1* and *egfl6* by *hnf1ba* and repression of *gbx1* by *hoxb1a* (Fig. 9). We also analyzed germline mutants for six genes (*gas6, gbx1, sall4, eglf6, celf2*, and *greb1l)*, but we do not detect hindbrain defects in any of the mutants. However, transcriptome profiling of *gas6* mutants identified differentially expressed genes involved in a variety of hindbrain related developmental processes – leading us to speculate that *gas6* may play subtle roles in hindbrain development. Thus, our study suggests that the regulatory logic differs in r4 versus r5/r6, but that both GRNs are relatively robust with a limited number of genes being absolutely required for their integrity.

### The r4 and r5/r6 gene regulatory networks operate by different mechanisms

Previous studies demonstrated that loss of PG1 *hox* function results in a mis-specified r4 [16, 24–26, 28, 29, 74, 75], leading us to hypothesize that all r4 genes are regulated by PG1 *hox* genes. Surprisingly, our ISH analysis of the r4 gene-set revealed continued expression in PG1 *hox* mutants. We note that, since PG1 *hox* mutants show defects in neuronal differentiation [24–26], *hoxb1a* and *hoxb1b* must regulate genes involved in neural differentiation. However, such genes are likely to be expressed in only a subset of cells in r4 and would not have been included in the r4 gene-set used for our analysis (which was restricted to genes expressed throughout r4). While a recent report indicates that expression of *fgf* pathway components is affected in *hoxb1b* mutants [26], we do not observe this, possibly due to differences in sensitivity of the ISH protocols used. Additionally, while our transcriptome analysis of *hoxb1b* mutants identified differentially expressed genes present in the hindbrain, the expression changes were relatively subtle. These results suggest that r4 gene regulation may require other factors in addition to PG1 *hox* genes. Indeed, we observe complete loss of expression of all tested r4 genes when *hoxb1b* and RA function is simultaneously disrupted, demonstrating that both factors are required to control r4 gene expression. Since RA signaling is unaffected by loss of PG1 *hox* function [26], these factors likely act in parallel (Fig. 9) – although it is unclear whether the combinatorial regulation by RA and *hoxb1b* acts directly at each r4 gene, or at an intermediary factor required to drive r4 gene expression. It is also unknown how RA signaling is initiated. A recent report concluded that RA signaling in the hindbrain is under control of *pbx* genes [26], but this effect is somewhat subtle, indicating that other as yet unknown factors may also control the RA pathway.

In r5/r6, the available data predict a relatively linear pathway where RA, FGF, *hnf1ba* and *valentino* control r5/r6 identity and disruption of any one of these factors causes r5/r6 defects. Additionally, at least in mice, r5 cells adopt an r6 fate in the absence of *krox20* [76, 77] and combined mutations in the mouse PG3 *hox* genes *Hoxa3* and *Hoxb3* result in loss of r5/r6 specific abducens motor neurons [78], suggesting that *krox20* and PG3 *hox* genes are also required for r5/r6 formation. In accordance with the prevailing model, we find that expression of all tested genes from the r5/r6 gene-set is abolished in *hnf1ba* and *valentino* mutants, as well as upon disruption of RA or FGF signaling. Notably, there are combinatorial interactions also in r5/r6 – for instance, *hnf1ba* and FGF act together to drive *valentino* expression [23, 30] – but the mechanism of combinatorial regulation differs between r4 and r5/r6. In r4, *hoxb1b* and RA function together to drive gene expression and either factor is sufficient to support expression. However, in r5/r6, neither *hnf1ba* nor FGF is sufficient to support r5/r6 gene expression. Hence, the r4 GRN appears less susceptible to disruptions than the r5/r6 GRN (Fig. 9). It is not clear why this would be the case, except that r4 is the earliest rhombomere to form and it acts as a key signaling center during hindbrain development, raising the possibility that there may have been greater evolutionary pressure to ensure that r4 forms properly.

### Repressive interactions may represent a key function of the hindbrain GRNs

Cross-talk between r4 and r5/r6 genes is a crucial part of establishing rhombomere boundaries and maintaining the uniqueness of each rhombomere. An example of this is seen in *hnf1ba* mutants where there is posterior expansion of the r4 genes *hoxb1a, fgf3,* and *efnb2a* into the mispatterned r5/r6 domain [23]. In this study, we identified *dusp6* (Fig. 1Civ), *spry1* (Fig. 1Div), and *eglf6* (Fig. 1Miv) as additional r4 genes whose expression domains are defined by *hnf1ba-*mediated repression. Importantly, *hnf1ba* is thought to act primarily as a transcriptional activator [79], raising the possibility that *hnf1ba* controls expression of a transcriptional repressor in r5/r6. Such an indirect effect may be mediated by *krox20*, which represses *Hoxb1* (murine ortholog of zebrafish *hoxb1a*) expression in r4 [80–84]. In particular, *krox20* activates the expression of Nab proteins, which are known negative regulators of transcription – making them possible candidates for mediating the effect of *hnf1ba* in repressing r4 gene expression [85]. Furthermore, the fact that only a subset of r4 genes is repressed by *hnf1ba*, suggests that additional factor (s) might be responsible for repressing the remaining r4 genes in an *hnf1ba*-independent manner.

We did not detect a reciprocal role for PG1 *hox* genes in repression of r5/r6 gene expression, but our experiments did demonstrate *hoxb1a*-mediated repression of *gbx1* in r4 (Fig. 3Hiii and Fig. 5Ai). We do not know the mechanism for this repression, but it may be indirectly mediated by Nlz proteins – members of a subfamily of zinc-finger proteins. Previous work demonstrated that Nlz proteins, which are found in r4, act as transcriptional repressors and *nlz* loss of function leads to gene expression from adjacent rhombomeres expanding into r4 [86–91]. Since *nlz1* expression is regulated by PG1 *hox* genes [87], *gbx1* repression may be indirectly mediated by *hoxb1a* via Nlz proteins.

### Members of the r4 and r5/r6 gene sets are not essential for hindbrain development

To test if members of the r4 and r5/r6 gene sets regulate caudal hindbrain formation, we analyzed germline mutants for six genes (*gas6, gbx1, sall4, eglf6, celf2*, and *greb1l*)*.* Our results reveal that in all of these mutant lines, hindbrain patterning and the subsequent development of the r4-specific Mauthner neurons, and the r5/r6-specific abducens neurons are normal. Indeed, detailed transcriptome analysis of *gas6* mutants identified differentially expressed genes involved in neuronal development, but the expression changes are subtle and cannot be detected by ISH. We cannot fully exclude the possibility that some residual gene activity persists in the specific mutants assayed. For instance, the *egfl6* mutation affects a splice junction and some mutants may also harbor maternal transcripts or proteins. However, in three cases (*gas6, sall4* and *greb1l*), we were able to assay the offspring of homozygous mutant parents, which eliminates the concern with maternal products. Furthermore, the viability of homozygous *gbx1* and *celf2* mutants was reduced, while *egfl6* homozygous mutants were infertile, demonstrating that these genes are important, just not for hindbrain development. Based on these analyses, it appears that most members of the r4 and r5/r6 gene sets may not be individually essential for hindbrain development. Accordingly, we recently found that *dusp6* and *dusp2* homozygous mutants also have normal hindbrain and neuronal pattering [55]. Hence, our data suggest that caudal hindbrain development is robust, and genes involved in this process most likely have redundant roles such that the loss of a single gene will not cause gross developmental defects. However, we note that these analyses are not exhaustive. In particular, previous genetic screens were not performed to saturation and most r4-r6 genes identified herein have not yet been tested by deletion in the germline. It therefore remains possible that additional key genes acting in r4-r6 will be identified.

## Conclusion

Gene regulatory networks are inherently complex, and this has been demonstrated in a variety of developmental processes in several model organisms. In this study we successfully positioned 22 previously uncharacterized genes into the existing GRN governing caudal hindbrain formation in the zebrafish (Fig. 9). Analysis of six mutant lines indicated that these genes are not absolutely required for r4-r6 formation but may have subtle roles. This leaves the previously reported factors RA, FGF, *hoxb1a, hoxb1b*, *hnf1ba* and *valentino* as key regulators of r4-r6 formation in the zebrafish. By extrapolation from work in the mouse [76–78], it is likely that *krox20* and PG3 *hox* genes also play a role in r4-r6 development in the zebrafish. While this may seem to be a small number of essential genes, there are other GRNs that have a limited number of core regulatory factors, like that of the transcriptional network regulating ES cells. Biochemical and bioinformatic studies done in both mice and humans show that Oct4, Sox2 and Nanog are the master regulators controlling the pluripotency and self-renewal of embryonic stem cells. While there are other TFs are involved in the larger embryonic stem cell GRN, they all feed into the core Oct4-Sox2-Nanong circuit [92, 93]. In support of the complex nature of GRNs, we demonstrate that regulation of r4 and r5/r6 is achieved via different mechanism. Specifically, our results support a novel model wherein r4 genes are under the combinatorial regulation of RA and *hoxb1b,* whereas r5/r6 genes are downstream of the previously described RA, FGF *hnf1ba* and *valentino* factors. We also identify novel interactions between the two gene-sets where the most striking observation is the repression of *gbx1* by *hoxb1a* in r4 (Fig. 9). In conclusion, our study demonstrates the distinct mechanisms of gene regulation in r4 and r5/r6 which stands as evidence to the complex nature of the GRN governing caudal hindbrain development in the zebrafish.

## Additional files

Additional file 1: **Figure S1.** Genotyping of embryos collected from cross of *hoxb1a* heterozygous parents. Several mutant lines used in this study are not viable as adults. As a result, many embryos used in assays were collected from crosses of heterozygous mutants. To ensure the presence of homozygous mutants in an assayed clutch, embryos were individually genotyped following ISH as outlined in the Methods section. Representative genotyping data for *hoxb1a* mutant embryos stained with (A) *spry1*, (B) *dusp6*, (C) *egfl6* and (D) *greb1l* demonstrate that approximately one quarter of the embryos assayed are homozygous mutant (indicated with asterisks), while 100% of the clutch showed normal staining for the assayed gene. (PDF 967 kb)

Additional file 2: **Table S1.** Identification of additional r4, r5 and r6 genes. This file represents the data downloaded from ZFIN and how it was parsed to generate a list of 107 rhombomere-restricted genes expressed in r4, r5 and r6. Sheet 1 lists 1820 genes that are expressed in the hindbrain, rhombomeres 4, 5 and 6. Sheet 2 represents 1194 genes that are expressed in the hindbrain, rhombomeres 4, 5 and 6 during the first 24 h development. Sheet 4 has the list of 107 genes that re restricted to rhombomeres 4, 5 and 6. Sheets 5, 6 and 7 represent the genes sorted according to their expression location and additional information associated to these genes are also listed in the last three sheets. (XLSX 112 kb)

Additional file 3: **Figure S2.** Expression of the r4 gene set is unaffected in *hoxb1a* mutants at least until 24hpf. Expression of *hoxb1a* (A), *meis1a* (B), *fgf3* (C) and *egfl6* (D) was assessed in wildtype (i) and *hoxb1a* mutant (ii) zebrafish at 24hpf. The black brackets mark r4 and dotted circles represent the otic vesicles (OV). (PDF 1051 kb)

Additional file 4: **Figure S3.** Generation and analysis of RNA-seq data from 18 hpf WT and *hoxb1b* mutant embryos. (A) Total RNA was collected from WT and *hoxb1b* mutant whole embryos and used for RNA-seq. (B) 866 differentially expressed genes were identified from RNA-Seq where seven of the 175 up-regulated genes and 78 of the 691 down-regulated genes are expressed in the hindbrain. (C) Top 20 GO terms for up-regulated and down-regulated genes. (D) A subset of genes was validated by RT-qPCR from independently collected samples. (PDF 589 kb)

Additional file 5: **Table S2.** Detailed analysis of WT and *hoxb1b* mutant RNA-seq data. 866 differentially expressed genes identified from the RNA-Seq experiment are listed in sheet 2. All GO terms associated with Biological Processes for both up-regulated and down-regulated genes are shown in sheets 3 and 4. Sheet 5 shows the subset of differentially expressed genes that is expressed in the hindbrain. In sheet 5, the genes in column A were derived from the ZFIN database (refer to sheet 3 in Additional file 2: Table S1). (XLSX 215 kb)

Additional file 6: **Figure S4.** Comparison between RNA-seq analyses of *Hoxa1* mutant mouse embryos and *hoxb1b* mutant zebrafish embryos. RNA-seq analysis of *Hoxa1* mutant mouse embryos was recently published in [48]. Comparing the mouse data set (A) with the 866 differentially expressed genes identified by our RNA-seq (B) revealed an overlap of 31 genes (C). Notably, none of these 31 genes has a rhombomere restricted expression pattern. (PDF 211 kb)

Additional file 7: **Figure S5.** SU5402 disrupts embryogenesis in *hoxb1b* mutants. Wildtype (i) and *hoxb1b* mutant (ii) zebrafish embryos were treated with SU5402 and assayed at various developmental stages by brightfield microscopy (A, B, F, H), or ISH to detect expression of *efnb2a/krox20* (C), *meis1a/krox20* (D), *irx7/krox20* (E) or *greb1l/krox20* (G). Note that defects in development are readily detectable in *hoxb1b* mutants treated with 50uM SU5402 (Aii, Bii), but not in WT embryos treated with SU5402 (Ai, Bi), nor in *hoxb1b* mutants treated with DEAB (Biii). As a result of these severe developmental defects, *hoxb1b* mutant embryos treated with SU5402 showed no specific staining for the r4 genes tested. (PDF 853 kb)

Additional file 8: **Figure S6.** Genotyping data for *sall4, egfl6, celf2* and *greb1l* mutants. *sall4, egfl6, celf2* and *greb1l* mutants generated by TILLING were procured from ZIRC. In each case, the mutation introduces a single nucleotide change (A; red text) causing a premature stop codon, except for *egfl6* where the point mutation disrupts an essential splice site in exon 8. (B) Sequencing traces showing expected single nucleotide changes in each mutant line. (PDF 758 kb)

Additional file 9: **Data S1.** Amino acid sequences of wildtype and mutant *gas6* alleles. Amino acid sequences of four mutant *gas6* alleles (um296, um297, um298, um299) aligned to the wildtype sequence shows that all four mutant alleles code for a premature stop codon after 96 amino acids. (DOCX 18 kb)

Additional file 10: **Figure S7.** Scheme for generating *gbx1* mutant line. (A) Schematic showing the 20 nucleotide (orange text) target site in exon 1 of *gbx1*. CCT represents the PAM sequence (blue box) and ATG (green box) is the start codon. Hpy188III target sequence is represented by the dotted red line, the red arrow denotes the cut site. (B) sgRNA and Cas9 mRNA was injected into 1-cell stage embryos. Injected embryos were raised to 24hpf and genomic DNA extracted from a pool of embryos. Hpy188III digest of PCR products amplified from genomic DNA (extracted from injected embryos) reveal the presence of a mutation (red boxes in gel). (C) Injected embryos were raised to give rise to F0 adults. These fish were crossed with WT adults to raise the F1 generation. At 3 months age, genomic DNA was extracted from fin-clips of individual F1 fish and genotyped as described in panel B. (D) Sequencing of F1 genomic DNA revealed transmission of two different mutant alleles; one allele (um300) has a 26-nucleotide insertion (green text) and the second allele (um301) has a two-nucleotide deletion (orange dashes). The resulting amino acid sequence is shown in the form of grey (amino acid sequence identical to wildtype) and yellow (out of frame amino acid sequence) boxes. Both mutant alleles result in premature stop codons upstream of the homeodomain*.* (E) Hpy188III digest of PCR products amplified from genomic DNA (extracted from individual F2 embryos) reveal the absence of homozygous mutants. (PDF 772 kb)

Additional file 11: **Data S2.** Amino acid sequences of wildtype and mutant *gbx1* alleles. Amino acid sequences of two mutant *gbx1* alleles (um300 and um301) aligned to the wildtype sequence shows that the mutant alleles introduce premature stop codons. (DOCX 14 kb)

Additional file 12: **Table S3.** Detailed analysis of WT and *gas6* mutants RNA-seq data. 1590 differentially expressed genes were identified from the RNA-Seq experiment and is shown in sheet 2. All the GO terms associated with Biological Processes for both up-regulated and down-regulated genes are listed in sheets 3 and 4. Sheet 5 shows the subset of differentially expressed genes that are expressed in the hindbrain. (XLSX 889 kb)

## Abbreviations

A-P: Anterior-posterior
CNS: Central nervous system
CRISPR: Clustered Regularly Interspaced Short Palindromic Repeats
DE: Differentially expressed
DEAB: Diethylamino-benzaldehyde
Dpf: days post fertilization
ENU: N-ethyl-N-nitrosourea
ES: Embryonic stem
EST: Expressed sequence tag
FGF: Fibroblast growth factor
GRN: Gene regulatory network
hpf: Hours post fertilization
ISH: In situ hybridization
MHB: Mid-hindbrain boundary
MO: Morpholino
OPC: Oligodendrocyte progenitor cell
PG1: Paralog group 1
r: rhombomere
RA: Retinoic Acid
sgRNA: single-stranded guide RNA
TALEN: Transcription activator-like effector nuclease
TF: Transcription factor
TILLING: Targeted Induced Local Lesions
WT: Wildtype
ZFIN: The Zebrafish Information Network
ZFN: Zing finger nuclease
ZIRC: The Zebrafish International Resource Center
