# Supplementary material for: Analysis of novel caudal hindbrain genes reveals different regulatory logic for gene expression in rhombomere 4 versus 5/6 in embryonic zebrafish
Source: Neural Dev. 2018 Jun 26;13:13. doi: 10.1186/s13064-018-0112-y (PMC6020313; doi:10.1186/s13064-018-0112-y)
Supplement: Supplementary file 1 — Figure S1. Genotyping of embryos collected from cross of hoxb1a heterozygous parents. Several mutant lines used in this study are not viable as adults. As a result, many embryos used in assays were collected from crosses of heterozygous mutants. To ensure the presence of homozygous mutants in an assayed clutch, embryos were individually genotyped following ISH as outlined in the Methods section. Representative genotyping data for hoxb1a mutant embryos stained with (A) spry1, (B) dusp6, (C) egfl6 and (D) greb1l demonstrate that approximately one quarter of the embryos assayed are homozygous mutant (indicated with asterisks), while 100% of the clutch showed normal staining for the assayed gene. (PDF 967 kb) [file 13064_2018_112_MOESM1_ESM.pdf]

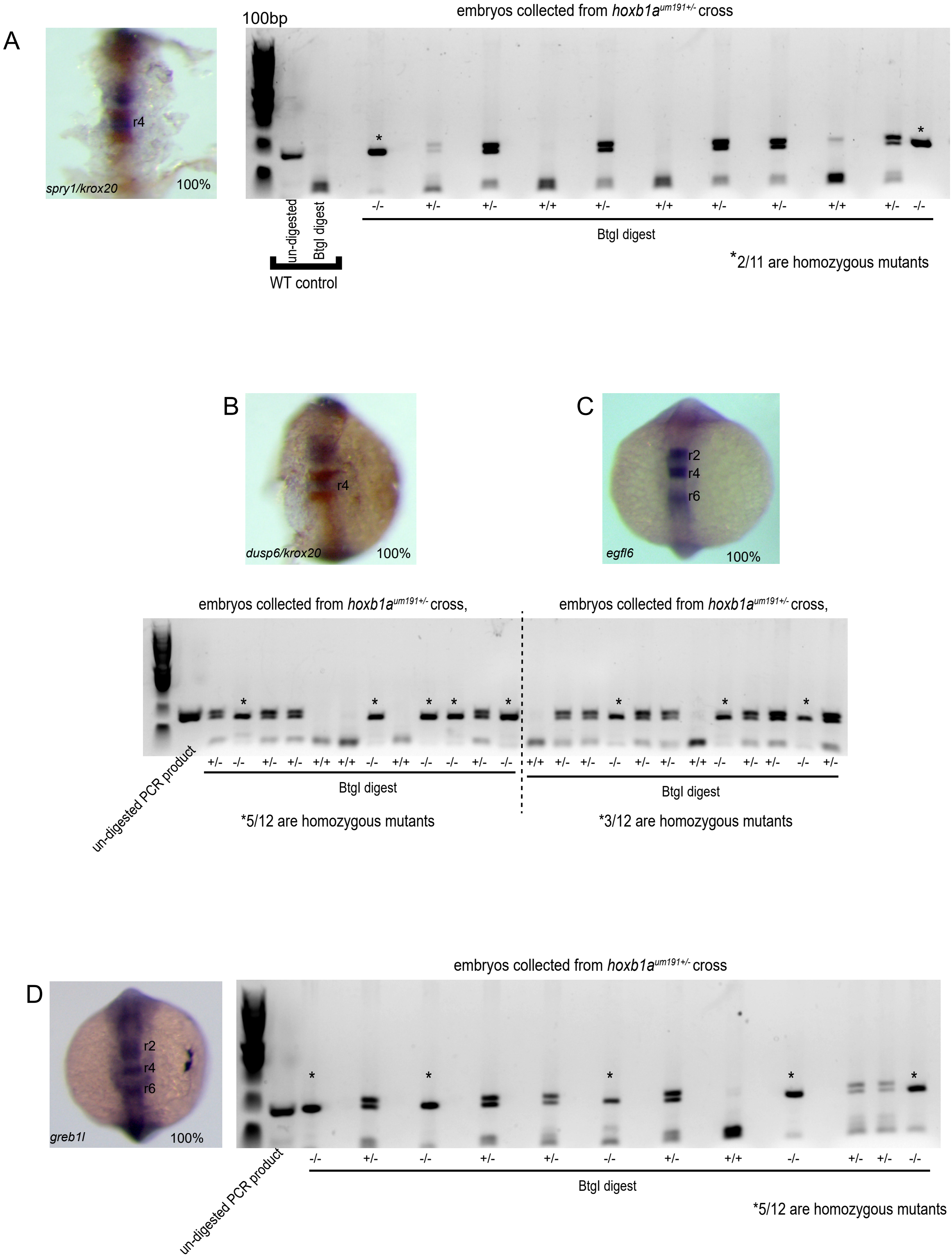
A
spry1/krox20
r4
100%
100bp
embryos collected from hoxb1a um191+/- cross
-/- +/- +/- +/+ +/- +/+ +/- +/- +/+ +/- -/-
un-digested
BtgI digest
WT control
BtgI digest
*2/11 are homozygous mutants
B
dusp6/krox20
r4
100%
C
egfl6
r2
r4
r6
100%
embryos collected from hoxb1a um191+/- cross,
+/- -/- +/- +/- +/+ +/+ -/- +/+ -/- -/- +/- -/-
BtgI digest
*5/12 are homozygous mutants
embryos collected from hoxb1a um191+/- cross,
+/+ +/- +/- -/- +/- +/- +/+ -/- +/- +/- -/- +/-
BtgI digest
*3/12 are homozygous mutants
un-digested PCR product
D
greb1l
r2
r4
r6
100%
embryos collected from hoxb1a um191+/- cross
-/- +/- -/- +/- +/- -/- +/- +/+ -/- +/- +/- -/-
BtgI digest
*5/12 are homozygous mutants
un-digested PCR product
