# Supplementary figures and images for: Analysis of novel caudal hindbrain genes reveals different regulatory logic for gene expression in rhombomere 4 versus 5/6 in embryonic zebrafish
Source: Neural Dev. 2018 Jun 26;13:13. doi: 10.1186/s13064-018-0112-y (PMC6020313; doi:10.1186/s13064-018-0112-y)

WT

*hoxb1a*<sup>-/-</sup>

A

24hpf  
*hoxb1a*

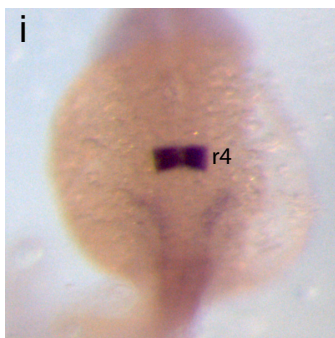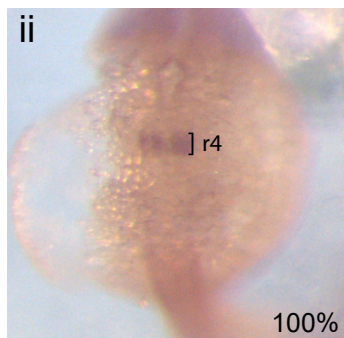

B

24hpf  
*meis1a*

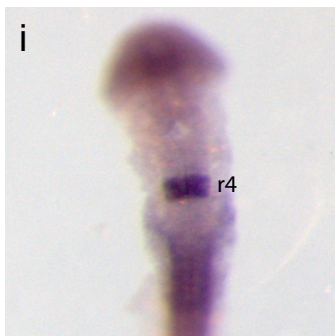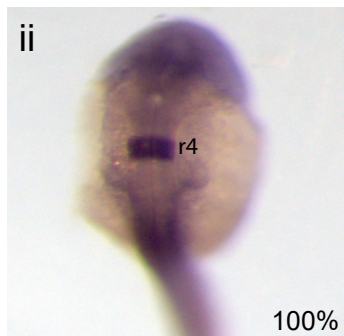

C

24hpf  
*fgf3*

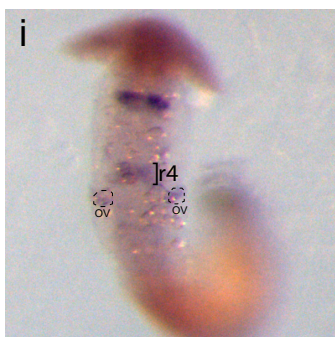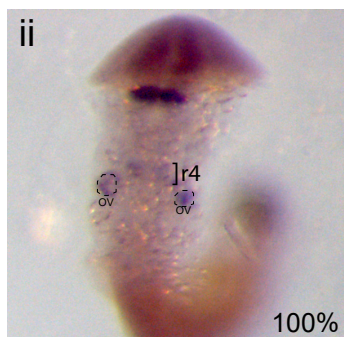

D

24hpf  
*egfl6*

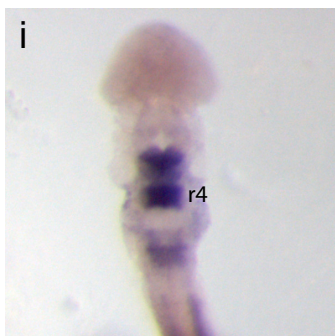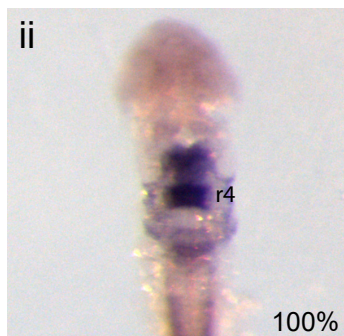

Supplement: Supplementary file 3 — Figure S2. Expression of the r4 gene set is unaffected in hoxb1a mutants at least until 24hpf. Expression of hoxb1a (A), meis1a (B), fgf3 (C) and egfl6 (D) was assessed in wildtype (i) and hoxb1a mutant (ii) zebrafish at 24hpf. The black brackets mark r4 and dotted circles represent the otic vesicles (OV). (PDF 1051 kb) [file 13064_2018_112_MOESM3_ESM.pdf]

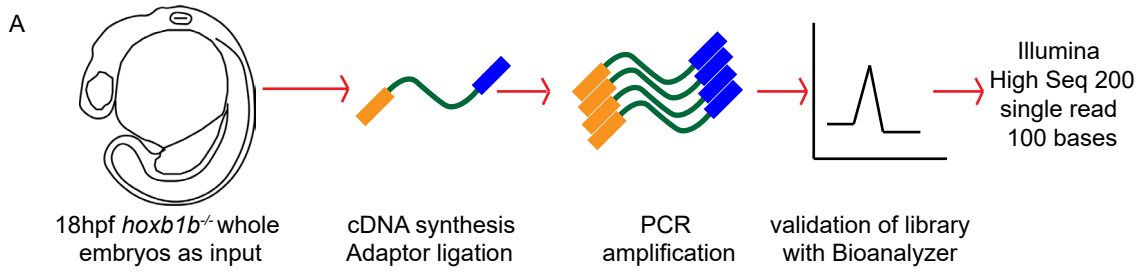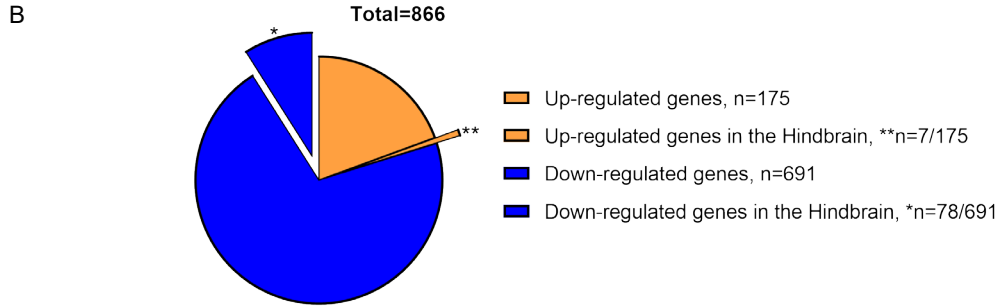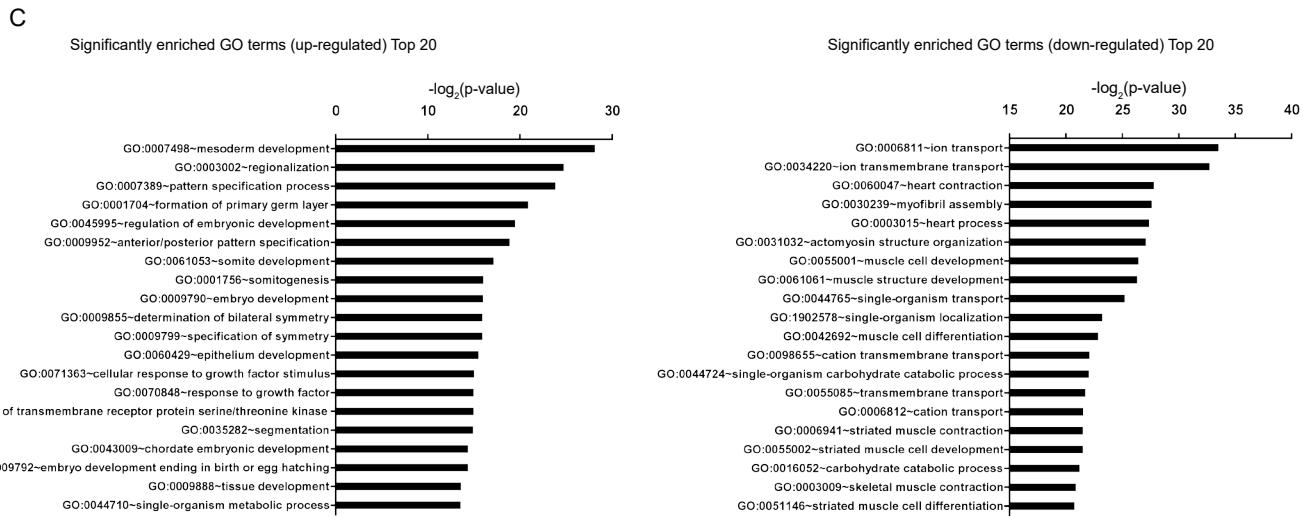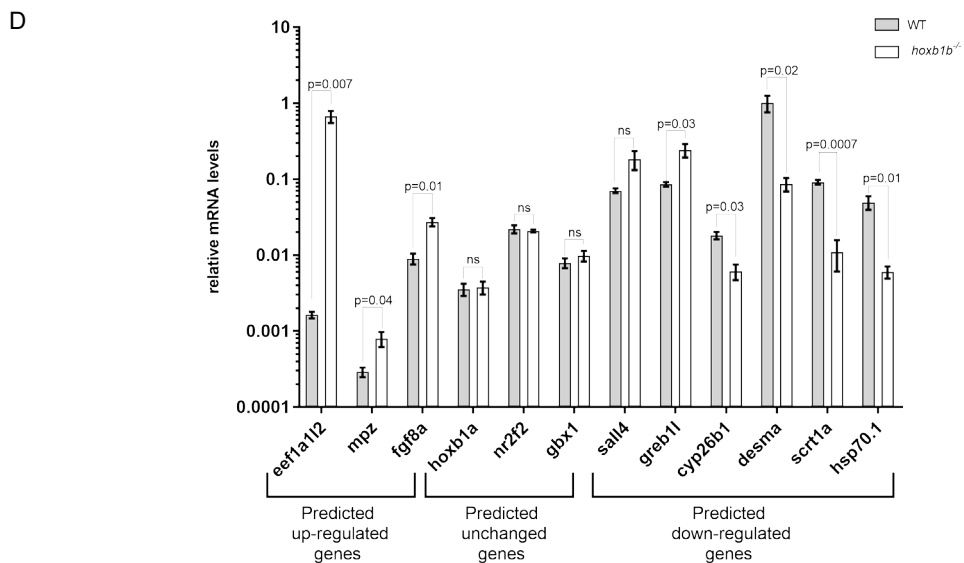

Supplement: Supplementary file 4 — Figure S3. Generation and analysis of RNA-seq data from 18 hpf WT and hoxb1b mutant embryos. (A) Total RNA was collected from WT and hoxb1b mutant whole embryos and used for RNA-seq. (B) 866 differentially expressed genes were identified from RNA-Seq where seven of the 175 up-regulated genes and 78 of the 691 down-regulated genes are expressed in the hindbrain. (C) Top 20 GO terms for up-regulated and down-regulated genes. (D) A subset of genes was validated by RT-qPCR from independently collected samples. (PDF 589 kb) [file 13064_2018_112_MOESM4_ESM.pdf]

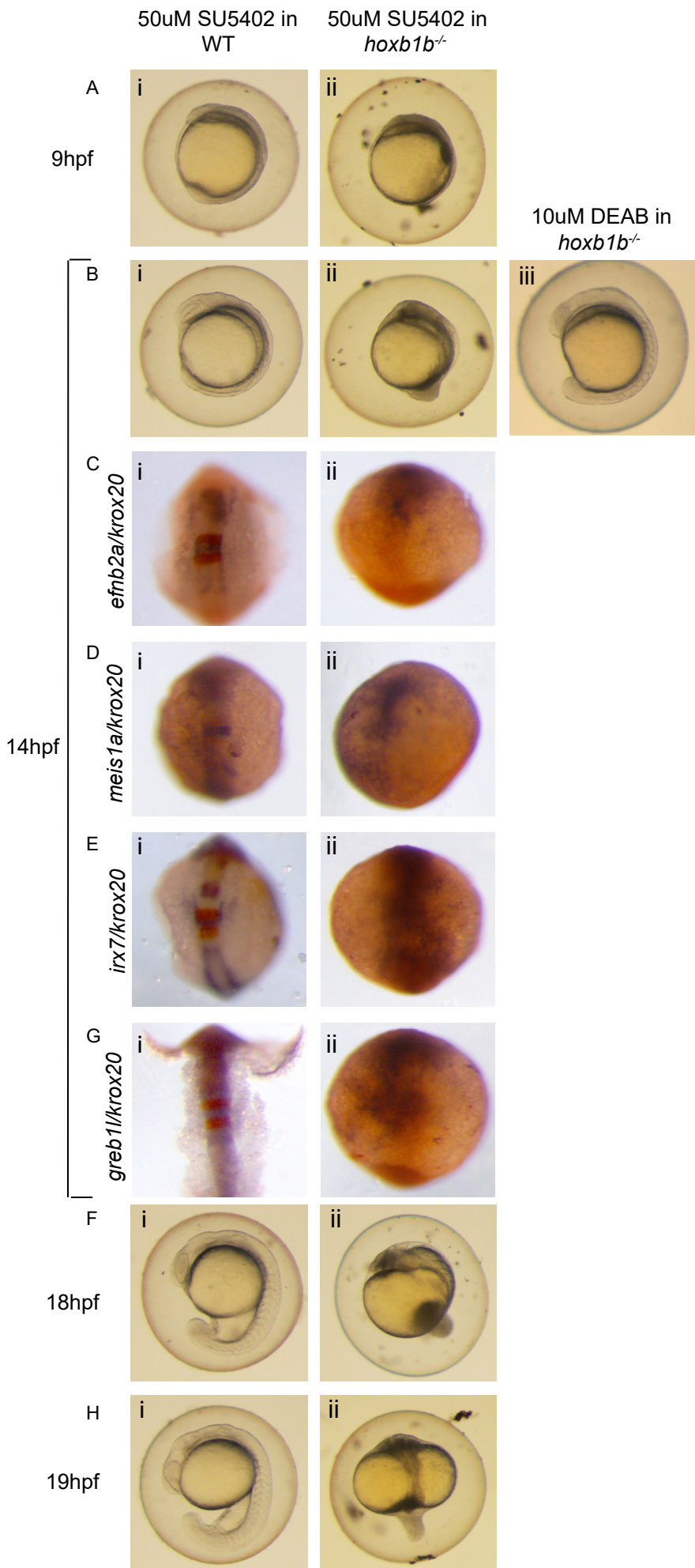

Supplement: Supplementary file 7 — Figure S5. SU5402 disrupts embryogenesis in hoxb1b mutants. Wildtype (i) and hoxb1b mutant (ii) zebrafish embryos were treated with SU5402 and assayed at various developmental stages by brightfield microscopy (A, B, F, H), or ISH to detect expression of efnb2a/krox20 (C), meis1a/krox20 (D), irx7/krox20 (E) or greb1l/krox20 (G). Note that defects in development are readily detectable in hoxb1b mutants treated with 50uM SU5402 (Aii, Bii), but not in WT embryos treated with SU5402 (Ai, Bi), nor in hoxb1b mutants treated with DEAB (Biii). As a result of these severe developmental defects, hoxb1b mutant embryos treated with SU5402 showed no specific staining for the r4 genes tested. (PDF 853 kb) [file 13064_2018_112_MOESM7_ESM.pdf]

A

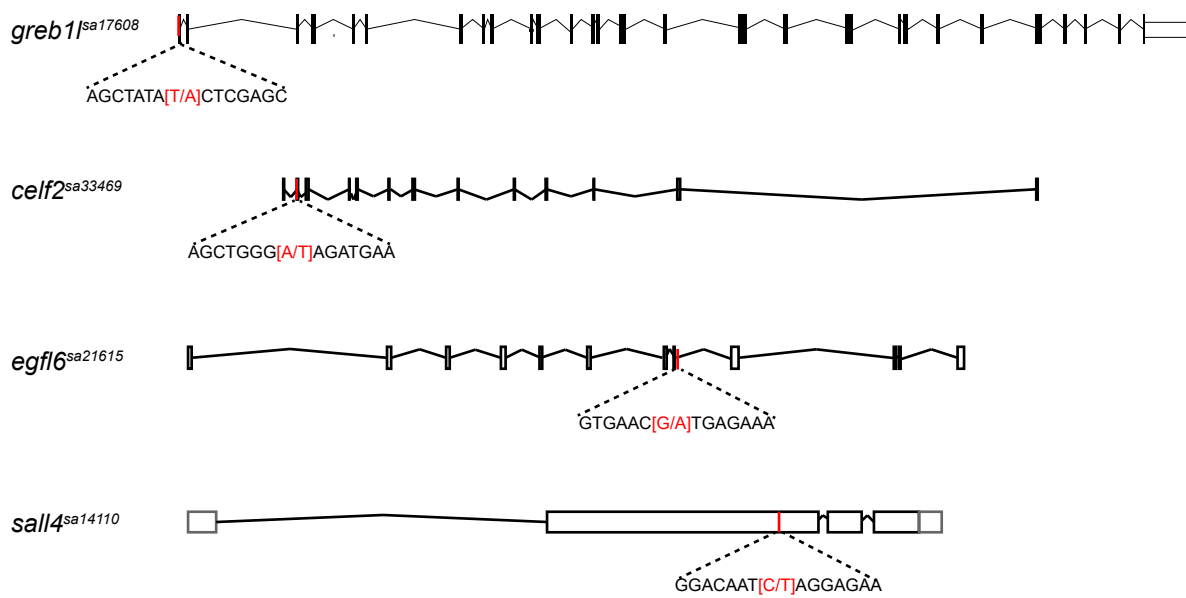

B

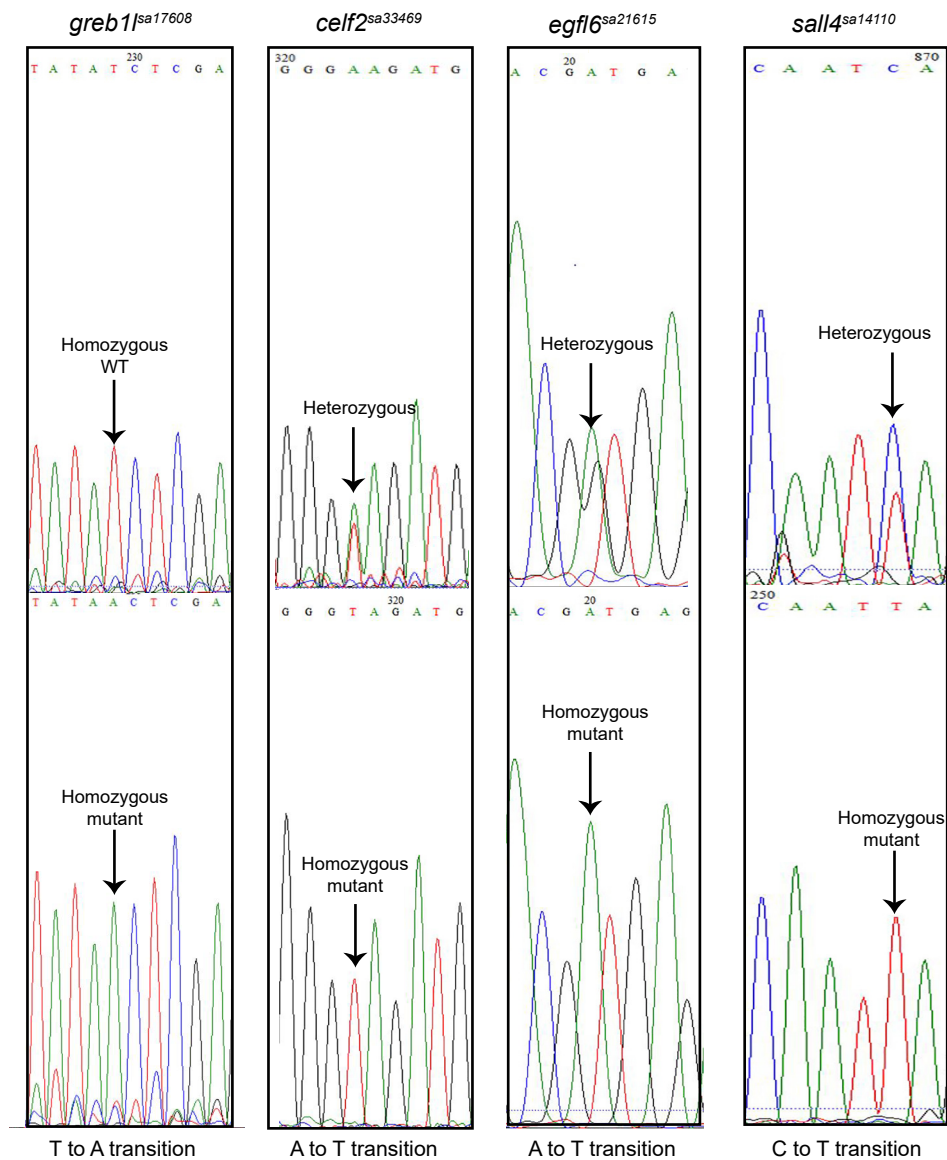

Supplement: Supplementary file 8 — Figure S6. Genotyping data for sall4, egfl6, celf2 and greb1l mutants. sall4, egfl6, celf2 and greb1l mutants generated by TILLING were procured from ZIRC. In each case, the mutation introduces a single nucleotide change (A; red text) causing a premature stop codon, except for egfl6 where the point mutation disrupts an essential splice site in exon 8. (B) Sequencing traces showing expected single nucleotide changes in each mutant line. (PDF 758 kb) [file 13064_2018_112_MOESM8_ESM.pdf]

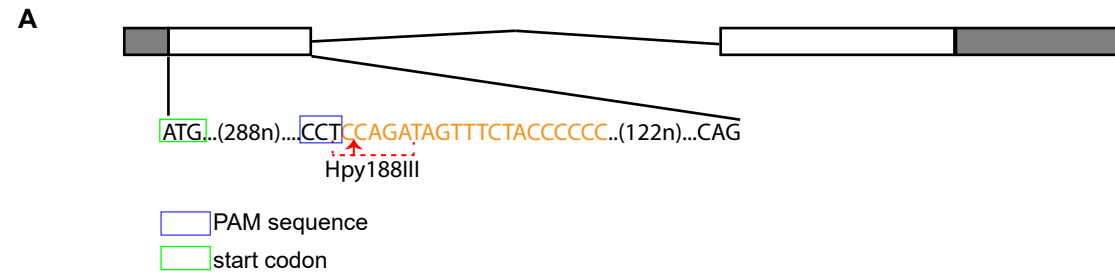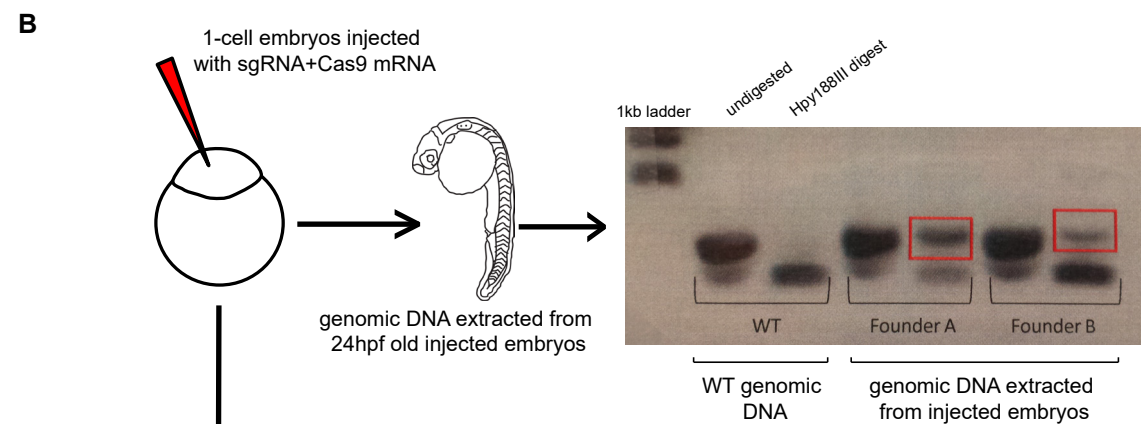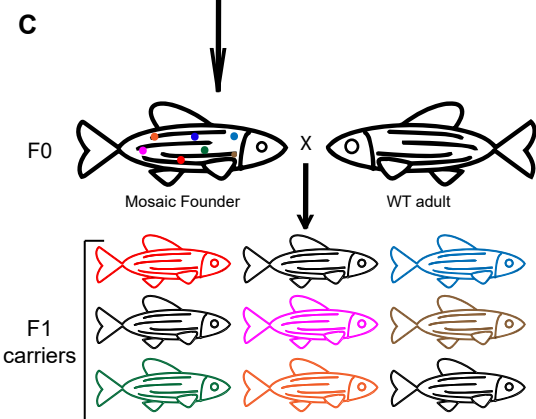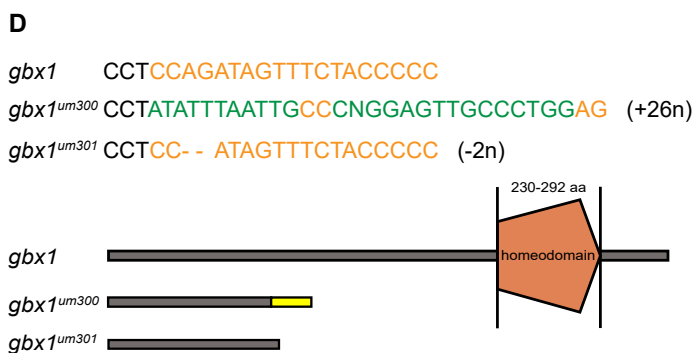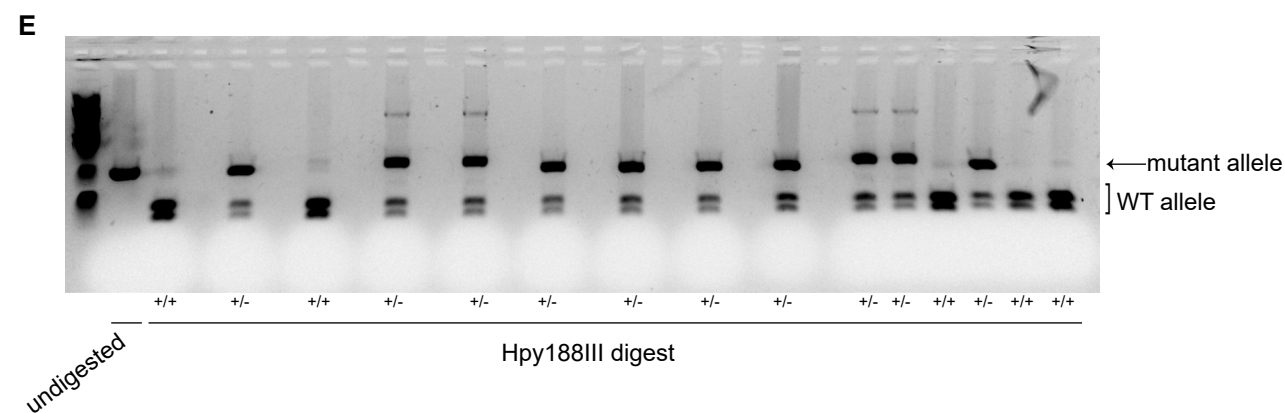

Supplement: Supplementary file 10 — Figure S7. Scheme for generating gbx1 mutant line. (A) Schematic showing the 20 nucleotide (orange text) target site in exon 1 of gbx1. CCT represents the PAM sequence (blue box) and ATG (green box) is the start codon. Hpy188III target sequence is represented by the dotted red line, the red arrow denotes the cut site. (B) sgRNA and Cas9 mRNA was injected into 1-cell stage embryos. Injected embryos were raised to 24hpf and genomic DNA extracted from a pool of embryos. Hpy188III digest of PCR products amplified from genomic DNA (extracted from injected embryos) reveal the presence of a mutation (red boxes in gel). (C) Injected embryos were raised to give rise to F0 adults. These fish were crossed with WT adults to raise the F1 generation. At 3 months age, genomic DNA was extracted from fin-clips of individual F1 fish and genotyped as described in panel B. (D) Sequencing of F1 genomic DNA revealed transmission of two different mutant alleles; one allele (um300) has a 26-nucleotide insertion (green text) and the second allele (um301) has a two-nucleotide deletion (orange dashes). The resulting amino acid sequence is shown in the form of grey (amino acid sequence identical to wildtype) and yellow (out of frame amino acid sequence) boxes. Both mutant alleles result in premature stop codons upstream of the homeodomain. (E) Hpy188III digest of PCR products amplified from genomic DNA (extracted from individual F2 embryos) reveal the absence of homozygous mutants. (PDF 772 kb) [file 13064_2018_112_MOESM10_ESM.pdf]
