# Supplementary material for: Analysis of novel caudal hindbrain genes reveals different regulatory logic for gene expression in rhombomere 4 versus 5/6 in embryonic zebrafish
Source: Neural Dev. 2018 Jun 26;13:13. doi: 10.1186/s13064-018-0112-y (PMC6020313; doi:10.1186/s13064-018-0112-y)
Supplement: Supplementary file 6 — Figure S4. Comparison between RNA-seq analyses of Hoxa1 mutant mouse embryos and hoxb1b mutant zebrafish embryos. RNA-seq analysis of Hoxa1 mutant mouse embryos was recently published in [48]. Comparing the mouse data set (A) with the 866 differentially expressed genes identified by our RNA-seq (B) revealed an overlap of 31 genes (C). Notably, none of these 31 genes has a rhombomere restricted expression pattern. (PDF 211 kb) [file 13064_2018_112_MOESM6_ESM.pdf]

A. 1537 DE genes in *Hoxa1* mutant mice at 10.5dpc. *De Kumar et.al.,2017*

B. 866 DE genes in *hoxb1b* mutant zebrafish at 18hpf

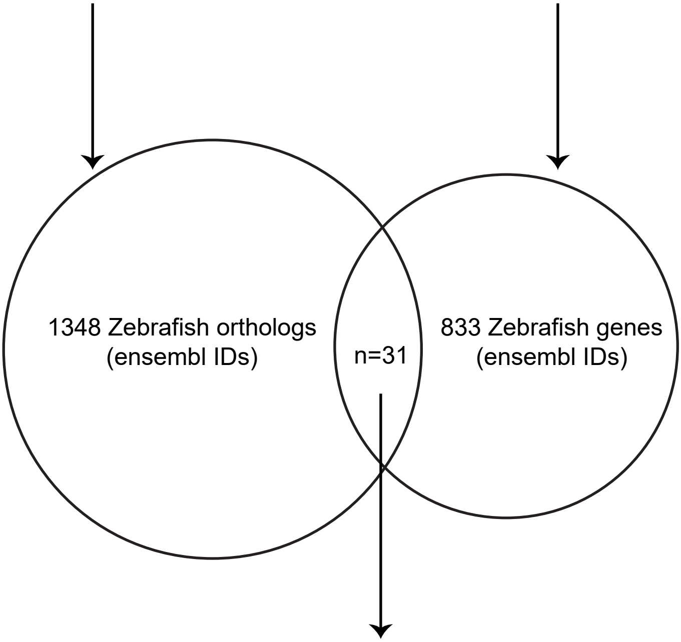

C

si:dkey-261m9.17(si:dkey-261m9.17)
serpin peptidase inhibitor, clade B (ovalbumin), member 1, like 4(serpinb1l4)
coronin, actin binding protein, 1A(coro1a)
B-cell CLL/lymphoma 11Aa(bcl11aa)
integrin beta 3a(itgb3a)
collagen, type I, alpha 1b(col1a1b)
follistatin b(fstb)
troponin C type 1b (slow)(tnnc1b)
xenotropic and polytropic retrovirus receptor 1a(xpr1a)
Rh blood group, D antigen(rhd)
glutamate receptor, ionotropic, AMPA 2b(gria2b)
SH3 domain binding glutamate-rich protein(sh3bgr)
glypican 1b(gpc1b)
aldolase a, fructose-bisphosphate, b(aldoab)
internexin neuronal intermediate filament protein, alpha b(inab)
insulin induced gene 1(insig1)
solute carrier family 25 (mitochondrial carrier; phosphate carrier), member 3a(slc25a3a)
collagen, type I, alpha 2(col1a2)
zgc:153405(zgc:153405)
calsequestrin 1a(casq1a)
fibrinogen alpha chain(fga)
myosin, light polypeptide 3, skeletal muscle(mylz3)
immunoglobulin superfamily containing leucine-rich repeat 2(islr2)
neurofilament, medium polypeptide a(nefma)
ceruloplasmin(cp)
prolactin(prl)
monoacylglycerol O-acyltransferase 2(mogat2)
stathmin-like 4, like(stmn4l)
premelanosome protein a(pmela)
NOC3-like DNA replication regulator(noc3l)
histone 1, H4, like (hist1h4l)
