# Supplementary material for: Analysis of novel caudal hindbrain genes reveals different regulatory logic for gene expression in rhombomere 4 versus 5/6 in embryonic zebrafish
Source: Neural Dev. 2018 Jun 26;13:13. doi: 10.1186/s13064-018-0112-y (PMC6020313; doi:10.1186/s13064-018-0112-y)
Supplement: Supplementary file 9 — Data S1. Amino acid sequences of wildtype and mutant gas6 alleles. Amino acid sequences of four mutant gas6 alleles (um296, um297, um298, um299) aligned to the wildtype sequence shows that all four mutant alleles code for a premature stop codon after 96 amino acids. (DOCX 18 kb) [file 13064_2018_112_MOESM9_ESM.docx]

**Supplemental Data 1: Protein sequences coded by *gas6* mutant alleles**

**WT M R E L V W S F C L V L L C S G F C S P V S V S S (1-25aa)**

**um296 M R W C G A S A S S C C V R A S A L P C P C P R A (1-25aa)**

**um297 M R G C G A S A S S C C V R A S A L P C P C P R A (1-25aa)**

**um298 M S W C G A S A S S C C V R A S A L P C P C P R A (1-25aa)**

**um299 M S W C G A S A S S C C V R A S A L P C P C P R A (1-25aa)**

**WT R Q A H Q F L R R T R R A N Q V F E E T K Q G H L (26-50aa)**

**um296 R P T S S C A E P A E P T R C S R R P N R A T W R (26-50aa)**

**um297 R P T S S C A E P A E P T R C S R R P N R A T W R (26-50aa)**

**um298 R P T S S C A E P A E P T R C S R R P N R A T W R (26-50aa)**

**um299 R P T S S C A E P A E P T R C S R R P N R A T W R (26-50aa)**

**WT E R E C V E E K C T K E E A R E V F E N D P E T E (51-75aa)**

**um296 G S V W R R S A L R R R R G K C S R M T R R R S I (51-75aa)**

**um297 G S V W R R S A L R R R R G K C S R M T R R R S I (51-75aa)**

**um298 G S V W R R S A L R R R R G K C S R M T R R R S I (51-75aa)**

**um299 G S V W R R S A L R R R R G K C S R M T R R R S I (51-75aa)**

**WT Y F Y P K Y Q A C M E R F G D S E K K K Q D L I T (76-100aa)**

**um296 S T P S I R L V W R D L G I Q R R R N R I Stop (76-96aa)**

**um297 S T P S I R L V W R D L G I Q R R R N R I Stop (76-96aa)**

**um298 S T P S I R L V W R D L G I Q R R R N R I Stop (76-96aa)**

**um299 S T P S I R L V W R D L G I Q R R R N R I Stop (76-96aa)**

**WT C V H N I P D Q C S P N P C Y H Y G T V R C E D K (101-125aa)**

**K G E F R C H C F T G W S G A T C Q N D V D E C I (126-150aa)**

**S G N G G C E H V C N N T M G S Y K C S C E D G Y (151-175aa)**

**R L S G H H S C L D V D E C V E T P D V C G S A H (176-200aa)**

**C S N L I G G L E C L C D E G F I Y D N I S R S C (201-225aa)**

**V D V D E C E T H V C E E E C V N T P G S F R C F (226-250aa)**

**C D G R L G K R L S S D M R S C E S I S L D R P L (251-275aa)**

**D M R R N S R S L Y L G R M F S G I P V V R L R F (276-300aa)**

**R R R V Q T G F T A E F D L R T F D P E G V I F F (301-325aa)**

**A G G H L N S S W I V L L V H H G K L E L Q L K Y (326-350aa)**

**G V V S R V T S S G P Q V N D G Q W H K I S V E E (351-375aa)**

**Q G R S L V I K I D R E A V M K I A V N G D L F T (376-400aa)**

**L N K N M H E L N L T V G G V P F R D D G L V S R (401-425aa)**

**V N P R L D G C M K D W R W L T G E D T S I Q E T (426-450aa)**

**I R H N E R M Q C Y A V E D H S A F Y P G H G F A (451-475aa)**

**Y F N H S H G D N Q T L S V H V T L R A A S S M G (476-500aa)**

**V L F A L V R Q D R V P F S I S L S D Y H P G T L (501-525aa)**

**Q W T K H V L V S L G D V V V G S V P V N L S D G (526-550aa)**

**Q T H T V N V T M S G N D S V L E V D A Q L A Q M (551-575aa)**

**E M M E G V D S L D L T S S Y S T F I G G I P D V (576-600aa)**

**S L V S S P V S A F F T G C M D V R V N G Q L L D (601-625aa)**

**V D E A Q H K H N D I R S H S C P L V D T L Q (626-648aa)**
