# Supplementary material for: Analysis of novel caudal hindbrain genes reveals different regulatory logic for gene expression in rhombomere 4 versus 5/6 in embryonic zebrafish
Source: Neural Dev. 2018 Jun 26;13:13. doi: 10.1186/s13064-018-0112-y (PMC6020313; doi:10.1186/s13064-018-0112-y)
Supplement: Supplementary file 11 — Data S2. Amino acid sequences of wildtype and mutant gbx1 alleles. Amino acid sequences of two mutant gbx1 alleles (um300 and um301) aligned to the wildtype sequence shows that the mutant alleles introduce premature stop codons. (DOCX 14 kb) [file 13064_2018_112_MOESM11_ESM.docx]

**Supplemental Data 2: Protein sequences coded by *gbx1* mutant alleles**

**WT M Q R P S G T G T A F S I D S L I G T P Q P R P G (1-25aa)**

**um300 M Q R P S G T G T A F S I D S L I G T P Q P R P G (1-25aa)**

**um301 M Q R P S G T G T A F S I D S L I G T P Q P R P G (1-25aa)**

**WT H L L Y T G Y P M F M P Y R P L M I P Q A L S H S (26-50aa)**

**um300 H L L Y T G Y P M F M P Y R P L M I P Q A L S H S (26-50aa)**

**um301 H L L Y T G Y P M F M P Y R P L M I P Q A L S H S (26-50aa)**

**WT S L P S G I P P L A P L A S F A G R L T N T F C A (51-75aa)**

**um300 S L P S G I P P L A P L A S F A G R L T N T F C A (51-75aa)**

**um301 S L P S G I P P L A P L A S F A G R L T N T F C A (51-75aa)**

**WT G L G Q G M P S M V A L T T T L P S F S D P P D S (76-100aa)**

**um300 G L G Q G M P S M V A L T T T L P S F S D P I F N (76-100aa)**

**um301 G L G Q G M P S M V A L T T T L P S F S D P P Stop(76-98aa)**

**WT F Y P P Q E M P G P R L G A D G T G M N R Q E S P (101-125aa)**

**um300 C X G V A L E I V S T P R R R C R D P G Stop (101-120aa)**

**WT H D E L K G S E L L N F T E T F Q A V A G E T K L (101-125aa)**

**Y S S D D E K L D L K A A E A A C S D R E D S S A (126-150aa)**

**D S E N E S F S D G N T C A S A S Q K G K L K G G (151-175aa)**

**S Q D A L P P G G S A G K S R R R R T A F T S E Q (176-200aa)**

**L L E L E K E F H C K K Y L S L T E R S Q I A H A (201-225aa)**

**L K L S E V Q V K I W F Q N R R A K W K R I K A G (226-250aa)**

**N V N N R S G E P V R N P K I V V P I P V H V N R (251-275aa)**

**F A V R S Q H Q Q I E P G S R P (276-316aa)**
